# Graphene related magnetic materials: micromechanical exfoliation of 2D layered magnets based on bimetallic anilate complexes with inserted [Fe^III^(acac_2_-trien)]^+^ and [Fe^III^(sal_2_-trien)]^+^ molecules†
†Electronic supplementary information (ESI) available: Experimental section, structural views of **1**, **2** and **3**, powder X-ray diffraction patterns of **1**, **2** and **3**, isothermal magnetization of **1**, **2** and **3** at 2 K, AC susceptibility measurements of **3**, DLS, EDS analysis, optical microscopy, SEM, TEM and AFM images of flakes of **2** and **4**. CCDC 1054372–1054374. For ESI and crystallographic data in CIF or other electronic format see DOI: 10.1039/c5sc00957j



**DOI:** 10.1039/c5sc00957j

**Published:** 2015-05-26

**Authors:** Alexandre Abhervé, Samuel Mañas-Valero, Miguel Clemente-León, Eugenio Coronado

**Affiliations:** a Instituto de Ciencia Molecular , Universidad de Valencia , Catedrático José Beltrán 2 , Paterna , 46980 , Spain . Email: miguel.clemente@uv.es ; Email: eugenio.coronado@uv.es ; Fax: +34 963543273 ; Tel: +34 963544405

## Abstract

The syntheses, structures and magnetic properties of the coordination compounds of formula [Fe^III^(acac_2_-trien)][Mn^II^Cr^III^(Cl_2_ An)_3_]·(CH_3_CN)_2_ (**1**), [Fe^III^(acac_2_-trien)][Mn^II^Cr^III^(Br_2_An)_3_]·(CH_3_CN)_2_ (**2**) and [Ga^III^(acac_2_-trien)][Mn^II^Cr^III^(Br_2_An)_3_]·(CH_3_CN)_2_ (**3**) are reported. They exhibit a 2D anionic network formed by Mn(ii) and Cr(iii) ions linked through anilate ligands, while the [Fe^III^(acac_2_-trien)]^+^ or [Ga^III^(acac_2_-trien)]^+^ charge-compensating cations are placed inside the hexagonal channels of the 2D network, instead of being inserted in the interlamellar spacing. Thus, these crystals are formed by hybrid layers assembled through van der Waals interactions. The magnetic properties indicate that these compounds behave as magnets exhibiting a long-range ferrimagnetic ordering at *ca.* 11 K, while the inserted Fe(iii) cations remain in the high-spin state. As for graphene, these layered materials can be exfoliated in atomically-thin layers with heights down to 2 nm by using the well-known Scotch tape method. Hence, this micromechanical procedure provides a suitable way to isolate ultrathin layers of this kind of graphene related magnetic materials. Interestingly, this method can also be successfully used to exfoliate the 2D anilate-based compound [Fe^III^(sal_2_-trien)][Mn^II^Cr^III^(Cl_2_An)_3_]·solv (**4**), which exhibits the typical alternated cation/anion layered structure. This result shows that the micromechanical exfoliation method, which has been extensively used for exfoliating van der Waals layered solids, can also be useful for exfoliating layered coordination compounds, even when they are formed by ionic components.

## Abstract 2

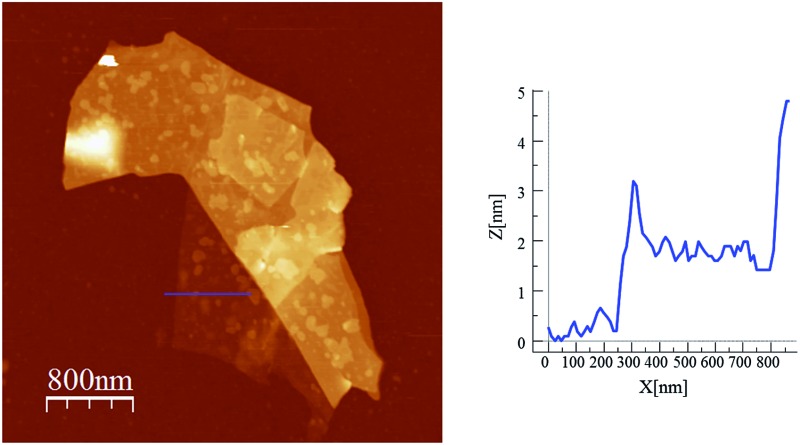
The Scotch tape method has been used for the exfoliation of layered coordination compounds formed by a 2D bimetallic anilate-based anionic network and Fe(iii) cationic complexes placed between or within the layers.

## Introduction

The identification of graphene among mechanically-exfoliated graphite sheets and the discovery of its extraordinary electronic properties^1^ has constituted a major breakthrough in materials science.^2^ Recently, this research has been extended to other 2D layered materials such as metal dichalcogenides, transition metal oxides or layered double hydroxides (LDHs). Owing to their unique morphology and properties, 2D materials have found a variety of applications in electronics, gas storage or separation, catalysis or high performance sensing.^2,3^ In general, these solids shear a layered structure with strong intralayer covalent bonding and weaker van der Waals or ionic interactions between the layers. As a result, many of these 2D materials can be exfoliated into individual nanosheets by different methods that involve either mechanical or solvent-mediated exfoliation methods.^4^

Notice that the research in this area has been dominated by the study of 2D materials exhibiting conducting (graphene) or semiconducting properties (Mo and W dichalcogenides), while the study of 2D magnetic materials has been largely ignored so far. From a chemical point of view, these materials are examples of old solid-state layered compounds. With the recent explosion of Metal–Organic Frameworks (MOFs), coordination chemistry has emerged as a source of new coordination polymers showing layered structures with, in some cases, interesting magnetic properties. The first attempt to exfoliate this type of materials started in 2008 with neutral carboxylate-based layered MOFs such as [Cu_2_Br(IN)_2_]_*n*_ (IN = isonicotinato)^5*b*^ or {Zn_2_(TPA)_4_(H_2_O)_2_·2DMF}_*n*_ (TPA = terephthalic acid).^5*c*^ These coordination polymers were exfoliated in 2D flakes of nanometric thickness (down to the monolayer level ∼1 nm) using solvent-mediated methods, mostly based on the sonication of crystals in a solvent.^5^ Exfoliation of other neutral 2D coordination polymers with 4-4′-bipyridine,^6^ pyrimidine,^7^ imidazole,^8^ peptidic^9^ or phosphonate^10^ bridging ligands were also reported later on. While these former studies were focused in the structural characterisation of the exfoliated nanosheets, more recent works have been focused on their properties and applications. Some examples include the nanomechanical characteristisation of MnDMS (DMS = 2,2-dimethyl-succinate) nanosheets,^5*d*^ the emission of a suspension of lanthanide organophosphonate nanosheets,^10*a*^ the enhanced adsorption capability to the Pb(ii) ions in aqueous solution of a exfoliated layered Cu(ii) phosphonate compared with that of the bulk material,^10*b*^ the photoluminescence and mechanical properties of few layer flakes of [Cu(μ-pym_2_S_2_)(μ-Cl)]_*n*_ (pymS_2_ = dipyrimidindisulde),^11^ or the preparation of ultrathin molecular sieve membranes with high H_2_ permeance and H_2_/CO_2_ selectivity from a suspension of Zn_2_(bim)_4_ (bim = benzimidazole) nanosheets.^8^

To our knowledge, examples of exfoliation of magnetic layered coordination polymers are scarce as many of them involve diamagnetic Zn^2+^ ions or long bridging ligands, which lead to weak magnetic interactions. Some of the few examples of magnetic 2D coordination polymers exfoliated so far are the Co^2+^ or Mn^2+^ 2,2-dimethylsuccinate frameworks reported by Cheetham *et al.*, which present antiferromagnetic ordering in the bulk.^5*f*^ However, there are examples of layered coordination polymers that can behave as ferro- or ferrimagnets at relatively high temperatures, which do not contain neutral magnetic networks. The archetypical example is provided by the family of layered oxalate-based bimetallic complexes. Its structure is formed by a 2D magnetic network of general formula [M^II^M^III^(C_2_O_4_)_3_]^–^ which can host a wide variety of functional cations in the interlamellar space.^12^ Thus, this family has provided unique examples of multifunctional materials combining cooperative magnetism with paramagnetism,^13^ photochromism,^14^ electrical conductivity,^15^ proton conductivity,^12a,16^ ferroelectricity,^17^ chirality,^18^ spin-crossover,^19^ or single-molecule magnetic behavior.^20^

**Scheme 1:**
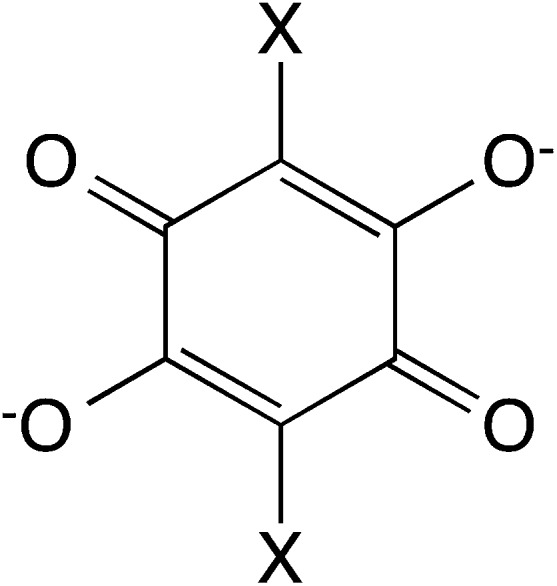
2,5-Dihydroxy-1,4-benzoquinone dianion derivatives used in this work.

In this work, we present the first attempt to exfoliate this type of hybrid coordination polymers. We have focused on the bimetallic complexes based on the anilate bis-bidentate bridging ligands, 2,5-dihydroxy-1,4-benzoquinone dianion and derivatives of formula C_6_O_4_X_2_^2–^ (X_2_An^2–^; X = Cl or Br, see Scheme 1).^21,22^ One of the advantages of this type of networks is that the bigger size of anilate ligands compared with oxalate ones may enable the insertion within the anion layer of the charge-compensating counter-cation, leading to a neutral layer that may be exfoliated using either mechanical or solvent-mediated exfoliation methods.

**Scheme 2:**
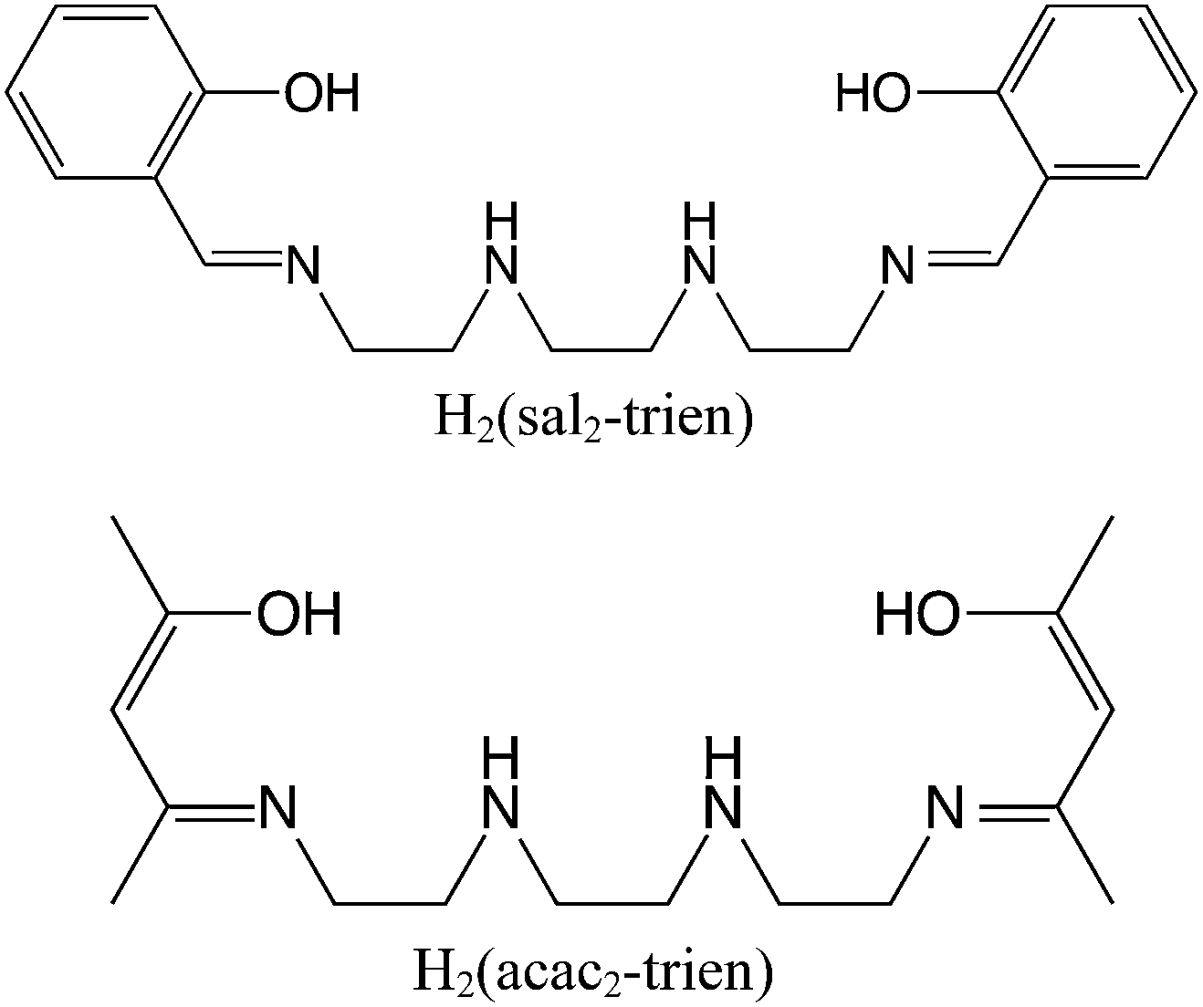
Ligands of the Fe(iii) complexes.

This is demonstrated in compounds [Fe^III^(acac_2_-trien)][Mn^II^Cr^III^(Cl_2_An)_3_]·(CH_3_CN)_2_ (**1**), [Fe^III^(acac_2_-trien)][Mn^II^Cr^III^(Br_2_An)_3_]·(CH_3_CN)_2_ (**2**) and [Ga^III^(acac_2_-trien)][Mn^II^Cr^III^(Br_2_An)_3_]·(CH_3_CN)_2_ (**3**) prepared in this work. These compounds contain the [M^III^(acac_2_-trien)]^+^ (M = Fe or Ga) complex, which is smaller in size than the previously used spin-crossover complex [Fe^III^(sal_2_-trien)]^+^ (see Scheme 2). This type of structure can be exfoliated using a micromechanical Scotch tape method leading to good quality micro-sheets of these layered magnets with lateral sizes of the order of microns and a thickness down to 2 nm, which roughly corresponds to a bilayer. The size of the sheets is significantly reduced (lateral sizes of tens of nm) when a solution-mediated exfoliation method is used. Finally, to check if the exfoliation is related to the neutral character of the layers, we have tried the micromechanical exfoliation of [Fe^III^(sal_2_-trien)][Mn^II^Cr^III^(Cl_2_An)_3_]·(CH_2_Cl_2_)_0.5_·(CH_3_OH)·(H_2_O)_0.5_·(CH_3_CN)_5_ (**4**), whose structure is formed by alternating layers of the anilate-based anionic network and [Fe^III^(sal_2_-trien)]^+^.^22^ Surprisingly, this coordination compound can also be easily exfoliated, despite the fact that the dominant interlayer interactions are now electrostatic. This result suggests that the micromechanical exfoliation method can be used, not only for exfoliating van der Waals solids formed by neutral layers, but also for exfoliating hybrid salts formed by charged layers.

## Results and discussion

### Synthesis

The method to prepare **1**, **2** and **3** is similar to that used to prepare other 2D anilate-based networks of Fe(iii) Schiff-base complexes.^22^ The best results were obtained using a methanol/chloroform (**1**) or methanol/dichloromethane (**2** and **3**) mixture to dissolve the Fe(iii) or Ga(iii) complex and Mn^2+^ salt, and acetonitrile to dissolve the anilate precursor. The chemical composition of these compounds, checked by microanalysis, shows a Fe/Mn/Cr/Cl (**1**), Fe/Mn/Cr/Br (**2**) or Ga/Mn/Cr/Br (**3**) ratio of 1/1/1/6. Elemental analyses confirm the purity of these samples, although the lower content of N and C in **1** and **3** could indicate the partial evaporation of CH_3_CN solvent molecules after extracting the crystals from the mother liquor. **4** was prepared following the previously reported procedure.^22^

### Structure

**Fig. 1:**
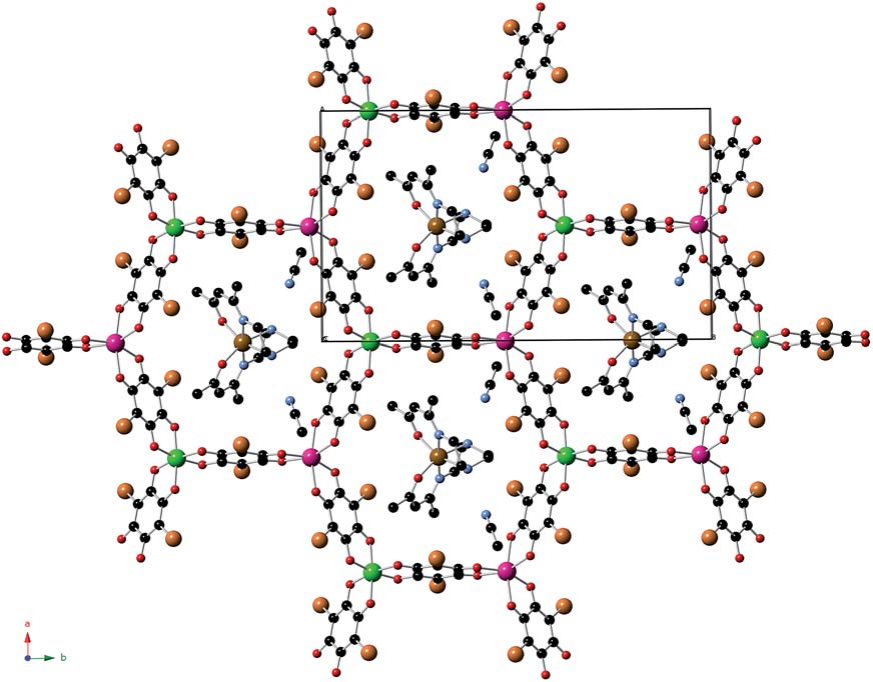
Projection of **2** in the *ab* plane (Fe (brown), Cr (green), Mn (pink) C (black), N (blue), O (red), Br (orange)). Hydrogen atoms have been omitted for clarity.

**1**, **2** and **3** crystallize in the monoclinic space group *C*2/*c*. The structure is formed by anionic 2D anilate-based layers in the *ab* plane of formula [Mn^II^Cr^III^(Cl_2_An)_3_]^–^ (**1**) or [Mn^II^Cr^III^(Br_2_An)_3_]^–^ (**2** and **3**) with the well-known honeycomb structure, which is similar to that reported in another anilate-based compounds such as **4** (Fig. 1 and S1 in the ESI†).^21,22^ It consists in a hexagonal layer where the Cr(iii) and Mn(ii) ions occupy alternating vertices of the hexagons and are linked through X_2_An bridges in such a way that each Mn(ii) is surrounded by three neighbouring Cr(iii) and *vice versa*. It contains crystallographically independent Mn and Cr ions with occupancies of 0.5 and characteristic Mn–O and Cr–O distances (see ESI†). Within the honeycomb layer, the bridged Mn(ii) and Cr(iii) present the opposite chirality, as expected. Due to the centrosymmetric character of the structure, Mn(ii) and Cr(iii) present the opposite configuration in neighbouring layers. These layers are alternated due the C-type unit cell that gives rise to two possible dispositions of the neighbouring layers (Fig. S2 in the ESI†).

**Fig. 2:**
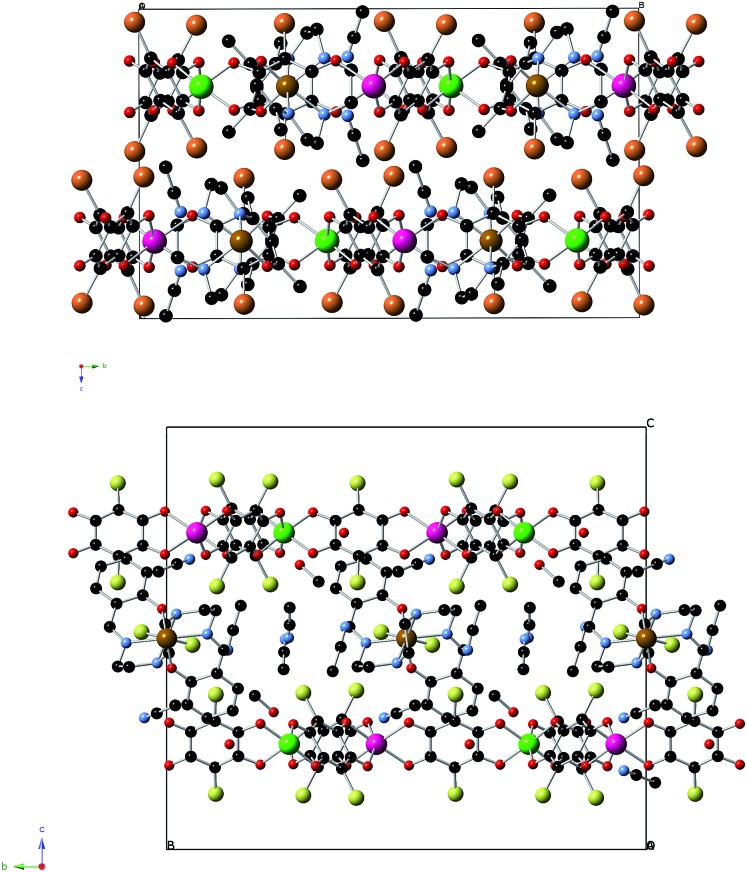
Projection of **2** (top) and **4** (bottom) in the *bc* plane (Fe (brown), Cr (green), Mn (pink) C (black), N (blue), O (red), Br (orange), Cl (yellow)). Hydrogen atoms have been omitted for clarity.

The main novelty of these structures is that the [Fe^III^(acac_2_-trien)]^+^ or [Ga^III^(acac_2_-trien)]^+^ templating cations are inserted into the hexagons of the 2D anilate-based network and not between the layers as in the previous oxalate or anilate-based 2D compounds. Thus, the centre of the hexagons is occupied by a crystallographically independent [Fe^III^(acac_2_-trien)]^+^ or [Ga^III^(acac_2_-trien)]^+^ complex with an occupancy of 0.5 (Fig. 1 and S1 in the ESI†). This gives rise to an important decrease of the interlayer separation between the anilate-based layers (Fig. 2 and S3 in the ESI†) with minimum distances between metals of neighbouring layers (7.39 Å for **1**, 7.53 Å for **2** and 7.57 Å for **3**) much smaller than those reported in the compounds with NBu_4_^+^ (9.69 Å), [(H_3_O)(phz)_3_]^+^ (9.03–9.21 Å) or [Fe^III^(sal_2_-trien)]^+^ and derivatives (11.06–11.92 Å).^21,22^ A second consequence of the insertion of [Fe^III^(acac_2_-trien)]^+^ complexes in the honeycomb anilate-based network is the absence of pores in the structures. These pores are present in most of the structures obtained with [Fe^III^(sal_2_-trien)]^+^ and derivatives and, as they are filled with disordered solvent molecules, give rise to problems in the structural resolution, which are not present in **1**, **2** and **3**. Overall, these anilate-based layers with inserted [Fe^III^(acac_2_-trien)]^+^ complexes may be viewed as neutral layers that interact with each other *via* van der Waals interactions. Thus, in **2**, the shortest contacts between neighbouring layers involve Br atoms from Br_2_An ligand with Br atoms from another Br_2_An ligand and CH_2_ and CH_3_ groups from [Fe^III^(acac_2_-trien)]^+^ complexes of neighbouring layers. The weak nature of these interactions is important for the exfoliation of the layers (see below).

[Fe^III^(acac_2_-trien)]^+^ complexes present a distorted octahedral geometry with Fe–N and Fe–O distances and N/O–Fe–N/O angles (see ESI†) similar to those of other high-spin [Fe^III^(sal_2_-trien)]^+^ or [Fe^III^(acac_2_-trien)]^+^ complexes.^23^ This indicates that at the temperature of the structural resolution (120 K), Fe(iii) complexes are in the HS state in agreement with the magnetic properties (see below). [Fe^III^(acac_2_-trien)]^+^ or [Ga^III^(acac_2_-trien)]^+^ complexes present numerous short contacts with the anilate ligands of the same layer. On the other hand, they are well-isolated by the anilate-based layer. Finally, acetonitrile solvent molecules occupy the holes between these layers and form hydrogen bonds with the NH groups of [Fe^III^(acac_2_-trien)]^+^ or [Ga^III^(acac_2_-trien)]^+^. Furthermore, they present short contacts with O and C atoms from X_2_An ligands. Powder X-ray diffraction patterns of **1**, **2** and **3** at 300 K confirm the structure obtained from single crystal X-ray diffraction experiments (Fig. S4 in the ESI†) with small differences especially in the case of **3**, which could be attributed to the partial loss of solvent molecules (CH_3_CN) after extracting the crystals from the mother liquor.

### Magnetic properties

**Fig. 3:**
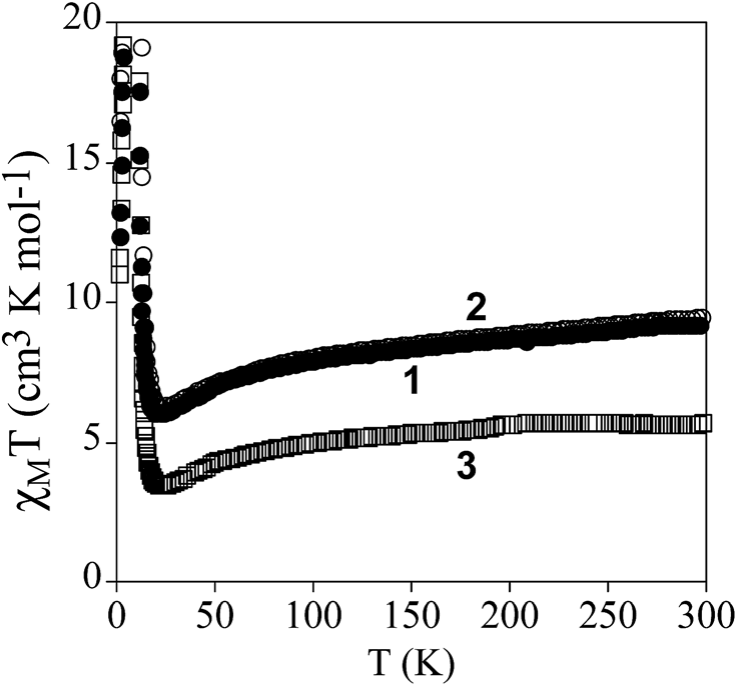
Temperature dependence of the product of the molar magnetic susceptibility times the temperature (*χ*_M_ *T*) of **1** (full circles), **2** (empty circles) and **3** (empty squares) with an applied field of 0.1 mT.

The product of the molar magnetic susceptibility times the temperature (*χ*_M_ *T*) of **1**, **2** and **3** is shown in Fig. 3. It presents at 300 K a value of 9.3 cm^3^ K mol^–1^ for **1** and 9.5 cm^3^ K mol^–1^ for **2**, which is close to the expected value for non-interacting Mn(ii) and Cr(iii) plus the contribution of a HS Fe(iii) ion (10.6 cm^3^ K mol^–1^ for *g* = 2). Furthermore, it is ∼3.7 cm^3^ K mol^–1^ higher than that of the reference compound **3**, which contains a diamagnetic [Ga^III^(acac_2_-trien)]^+^ in the place of [Fe^III^(acac_2_-trien)]^+^ (5.7 cm^3^ K mol^–1^). When the temperature is lowered, *χ*_M_ *T* shows a continuous decrease reaching a minimum at *ca.* 23 K of 6.0 cm^3^ K mol^–1^ for **1**, 6.3 cm^3^ K mol^–1^ for **2** and 3.4 cm^3^ K mol^–1^ for **3** followed by a sharp increase at lower temperatures with a maximum at *ca.* 8.8 K. The decrease of *χ*_M_ *T* with the temperature may be attributed to antiferromagnetic MnCr interactions mediated through the X_2_An^2–^ bridges, as observed in [NBu_4_]^+^, [(H_3_O)(phz)_3_]^+^ or [Fe^III^(sal_2_-trien)]^+^ salts containing similar [Mn^II^Cr^III^(X_2_An)_3_]^–^ layers (X = Cl, Br, I and H).^21,22^ Since the ground spin states of Cr(iii) and Mn(ii) are different (3/2 and 5/2, respectively), this interaction leads to an antiferromagnetic coupling that results in a *χ*_M_ *T* minimum, followed by an increase of *χ*_M_ *T* below *ca.* 20 K, and finally by a ferrimagnetic long range ordering at low temperatures for the three compounds. A comparison of the magnetic behaviour observed in **1** and **2** with that observed in the reference compound **3** shows that the difference of *χT* values stays almost constant in the range 300–25 K, confirming that in **1** and **2** the Fe(iii) is HS. A similar behaviour was observed for **4**.^22^

**Fig. 4:**
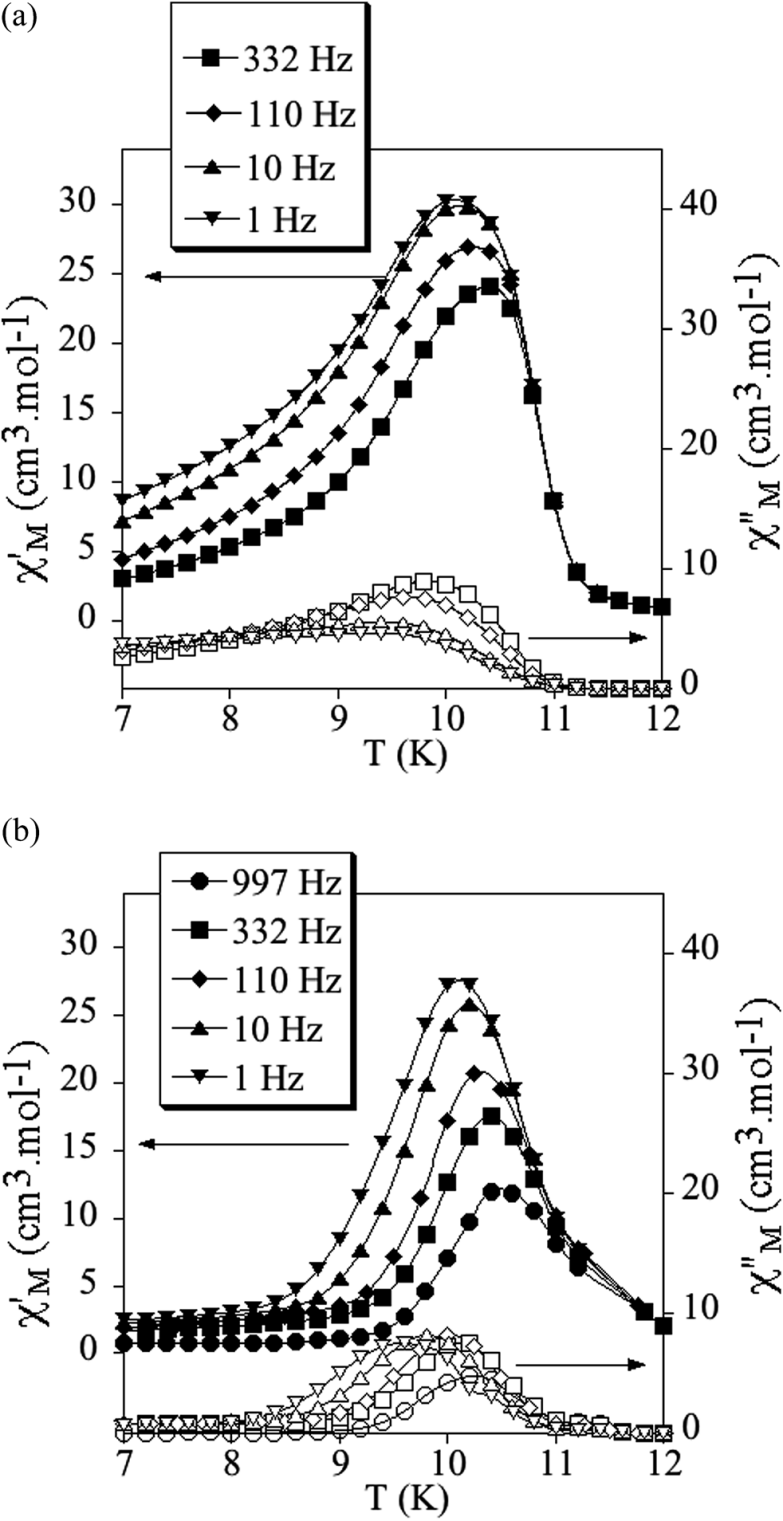
Temperature dependence of the in-phase AC susceptibility (*χ*′) (filled symbols) and the out-of-phase AC susceptibility (*χ*′′) (empty symbols) of **1** (a) and **2** (b).

The confirmation of the long-range order and a more accurate determination of the ordering temperatures are obtained from the susceptibility measurements performed with an alternating magnetic field (AC susceptibility). These measurements show a frequency-independent peak in the in phase molar susceptibility (*χ*′_M_) and out of phase molar susceptibility (*χ*′′_M_) for the three compounds (Fig. 4 and S5 in the ESI†). The *T*_c_, determined as the temperature at which *χ*′′_M_ becomes non-zero, is 10.8 K for **1**, 11.4 K for **2** and 11.6 K for **3**. These *T*_c_ values are close to those found for [Mn^II^Cr^III^(Cl_2_An)_3_]^–^ or [Mn^II^Cr^III^(Br_2_An)_3_]^–^ salts of [Fe^III^(sal_2_-trien)]^+^ and derivatives.^22^

The ferrimagnetic nature of the long range ordering is confirmed by the isothermal magnetization measurements at 2 K that show a sharp increase of the magnetization at low fields that becomes more gradual at higher fields (Fig. S6 in the ESI†). At low fields (*H* < 0.2 T), the magnetization of the three compounds increases with a high slope reaching values in the range 1.2–1.7 *μ*B at *H* = 0.1 T (inset in Fig. S6†). At higher fields, the magnetization of **1** and **2** shows a gradual and nonlinear increase (5.1 *μ*B for **1** and 5.0 *μ*B for **2** at 5 T), while that of **3** tends to saturation at lower values (1.8 *μ*B at 5 T). In **3**, this magnetization value is close to that expected for a ferrimagnetic Mn^II^Cr^III^ network (*M*_s_ = 5 *μ*B – 3 *μ*B = 2 *μ*B). For **1** and **2**, the major contribution to the gradual nonlinear increase observed at higher fields is due to the contribution of the paramagnetic HS Fe(iii). These isothermal magnetization measurements also provide an additional proof of the magnetic ordering exhibited by these compounds since they present hysteresis below the ordering temperatures with coercive fields of *ca.* 65 mT for **1**, 77 mT for **2** and 72 mT for **3** (inset in Fig. S6†).

### Exfoliation

**Fig. 5:**
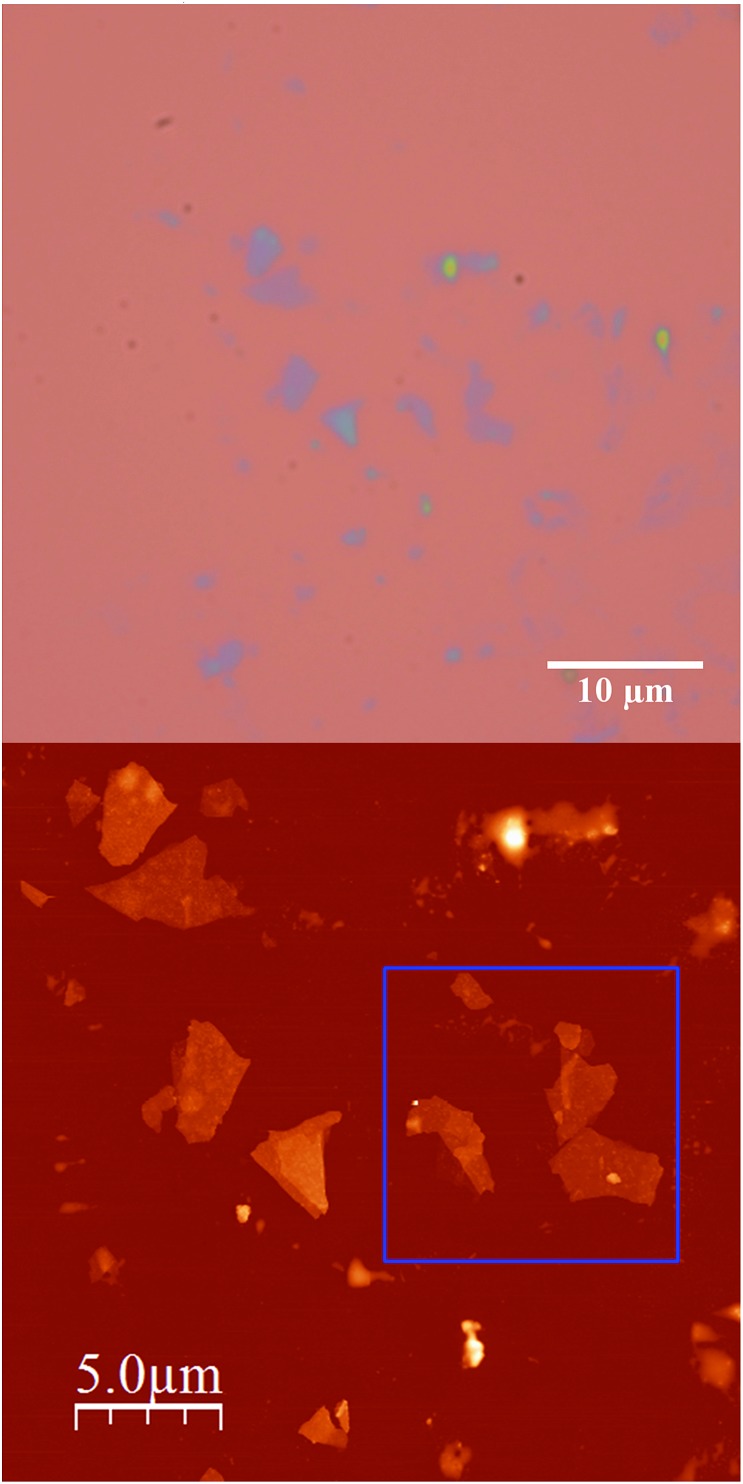
(a) Optical microscopy image (top) and AFM image of flakes of **2** obtained by mechanical exfoliation on a 285 nm SiO_2_/Si substrate taken in the same zone (bottom).

**Fig. 6:**
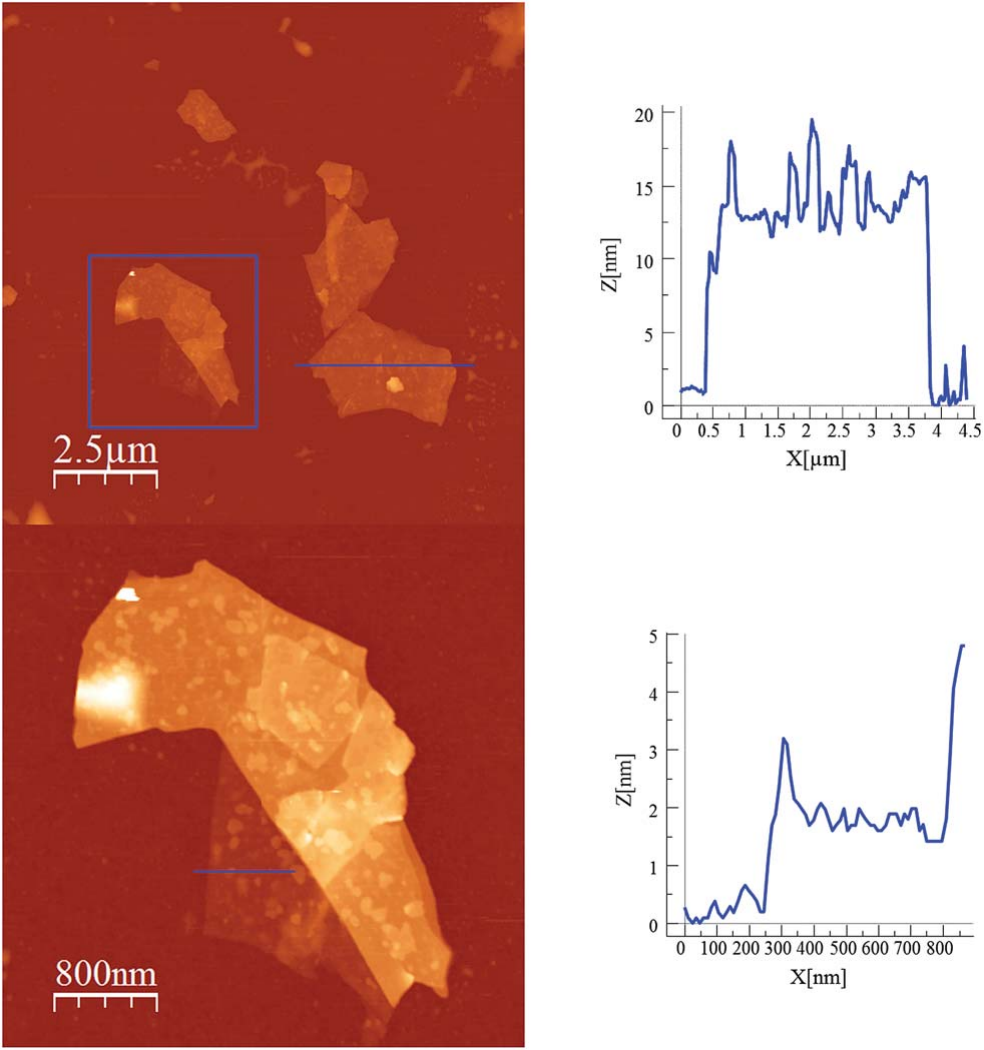
AFM images and height profiles of flakes of **2** shown in Fig. 5 obtained by mechanical exfoliation on a 285 nm SiO_2_/Si substrate.

Scanning electron microscopy (SEM) images of bulk crystals of **2** suggest their layered character (Fig. S7 in the ESI†). In order to exfoliate the layers, micromechanical and solvent-mediated methods were used. To date, the micromechanical exfoliation (the so-called Scotch tape method) is the most efficient way to produce the cleanest, highly crystalline and atomically thin microsheets of a layered material.^24^ To our knowledge it has never been applied to layered coordination polymers. In a typical mechanical exfoliation process, the 2D material is first peeled off from its bulk crystals by using adhesive Scotch tape. After the Scotch tape is removed, layers of the 2D material are left on the substrate. In crystals of **2** this procedure produced flakes with different sizes and thicknesses randomly distributed over the substrate. Interestingly, a small fraction of them presented heights of a few nm. These thin flakes were identified by the contrast difference between the flakes and the substrate in the colour image obtained with an optical microscope (Fig. 5). Microanalysis recorded during the SEM characterization indicated a Fe/Mn/Cr/Br ratio of 1/1/1/6 for the flakes, which is consistent with the initial composition of the crystals (Fig. S8 in the ESI†). However, it was not possible to evaluate the composition of the thinnest flakes due to the low sensitivity of this technique. AFM topography images revealed that the flakes of **2** display maximum lateral dimensions of ∼5 μm, with well-defined edges and angles (Fig. 5, 6 and S9 in the ESI†). The heights of the largest flakes of **2** are around 10–20 nm. On the other hand, smaller microsheets with heights of less than 2 nm were also found (Fig. 6 and S9 in the ESI†). The layered nature is reflected by the presence of terraces with different heights. Comparison of the thickness of the thinnest sheets with the layer thickness calculated from single crystal X-ray diffraction data (0.7 nm) is consistent with the presence of, at least, bilayers. Note that 2D materials are often raised by a few Angstrom above the supporting surface, probably because of a layer of contaminant (adsorbed water) between the flakes and the substrate.^8,25^

**Fig. 7:**
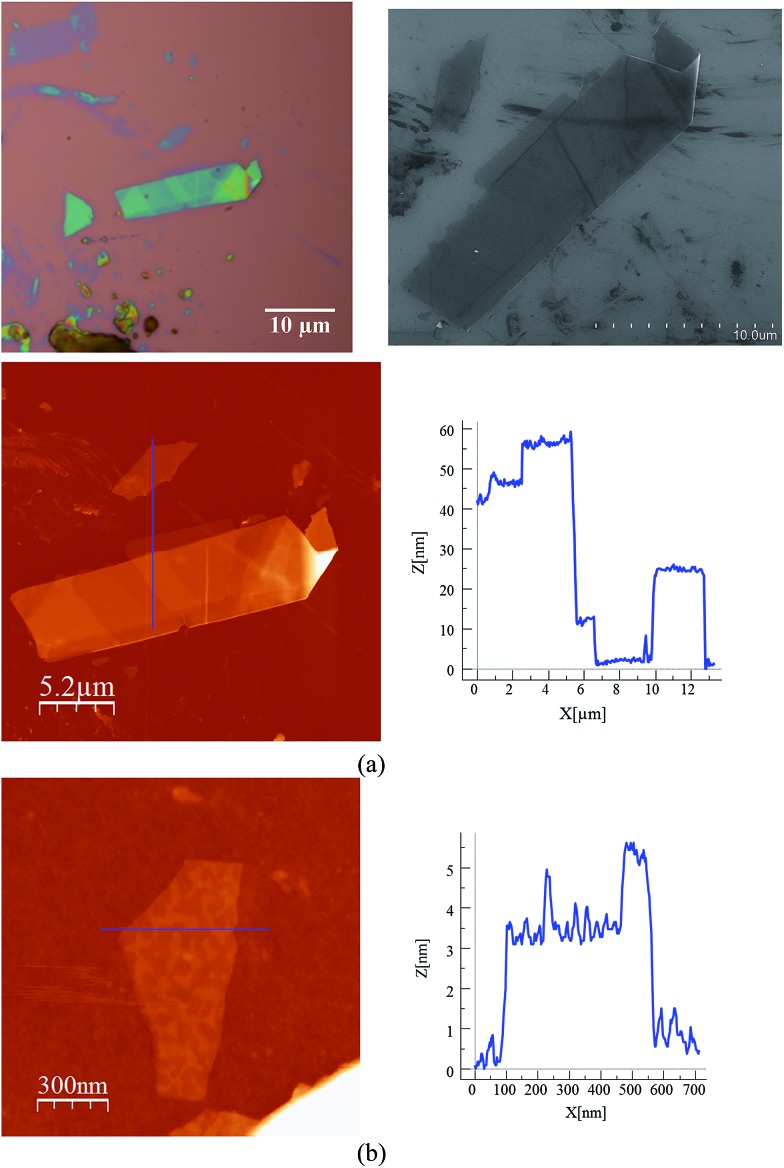
Optical microscopy image (top left), SEM image (top right) and AFM image with height profile of a flake of **4** obtained by mechanical exfoliation on a 285 nm SiO_2_/Si substrate taken in the same zone (a). AFM image with height profile of a flake of **4** (b).

**Fig. 8:**
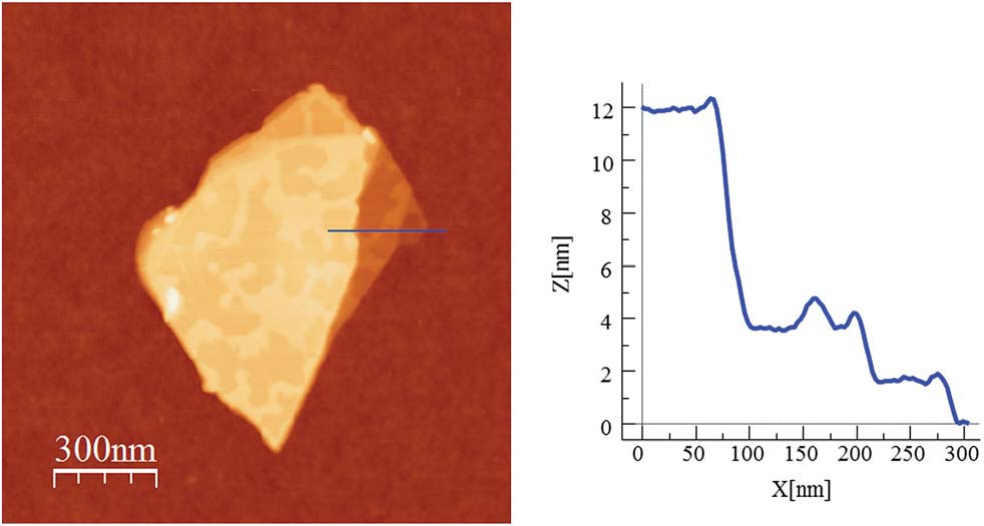
AFM image and height profile of a flake of **4** obtained by mechanical exfoliation on a 285 nm SiO_2_/Si substrate. Three nanosheets of about 2, 4 and 12 nm are discernible.

In a second step the same method was applied to a compound with a similar 2D network but formed by alternating anionic layers of the anilate-based network and a Fe(iii) cationic complex of larger size (compound **4**). We observe that, despite the segregated cation/anion layered structure showed by this salt, the layers can be exfoliated using a micromechanical procedure. Thus, rectangular flakes of larger lateral size than those isolated in compound **2** (up to 20 microns) can be obtained with well defined terraces and a minimum thickness of *ca.* 2 nm, which may correspond to that of a single cation/anion hybrid layer (≈1.2 nm; see Fig. 7, 8 and S10 in the ESI†). Microanalysis recorded during the SEM characterization of some of the flakes indicated a Fe/Mn/Cr/Cl ratio of 1/1/1/6, which is consistent with the initial composition of the crystals (Fig. S8 in the ESI†).

**Fig. 9:**
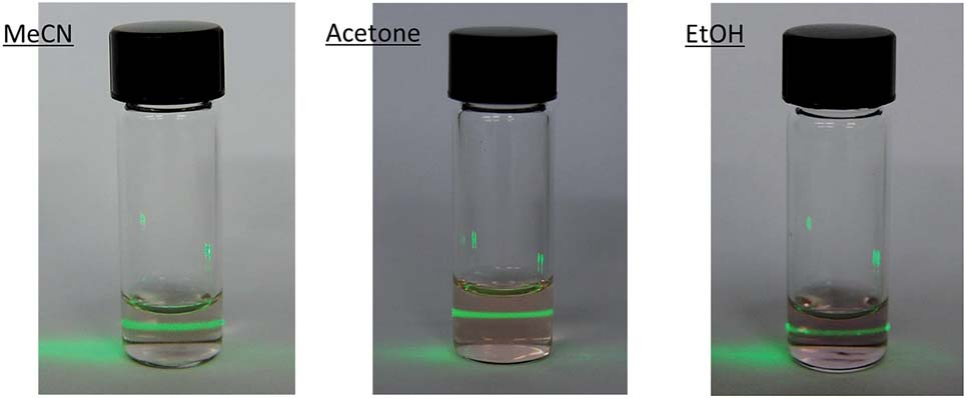
Tyndall effect of crystals of **2** after immersing in acetone, ethanol or acetonitrile (1.0 mg in 1 mL) overnight and then ultrasonicating for 1 minute.

**Fig. 10:**
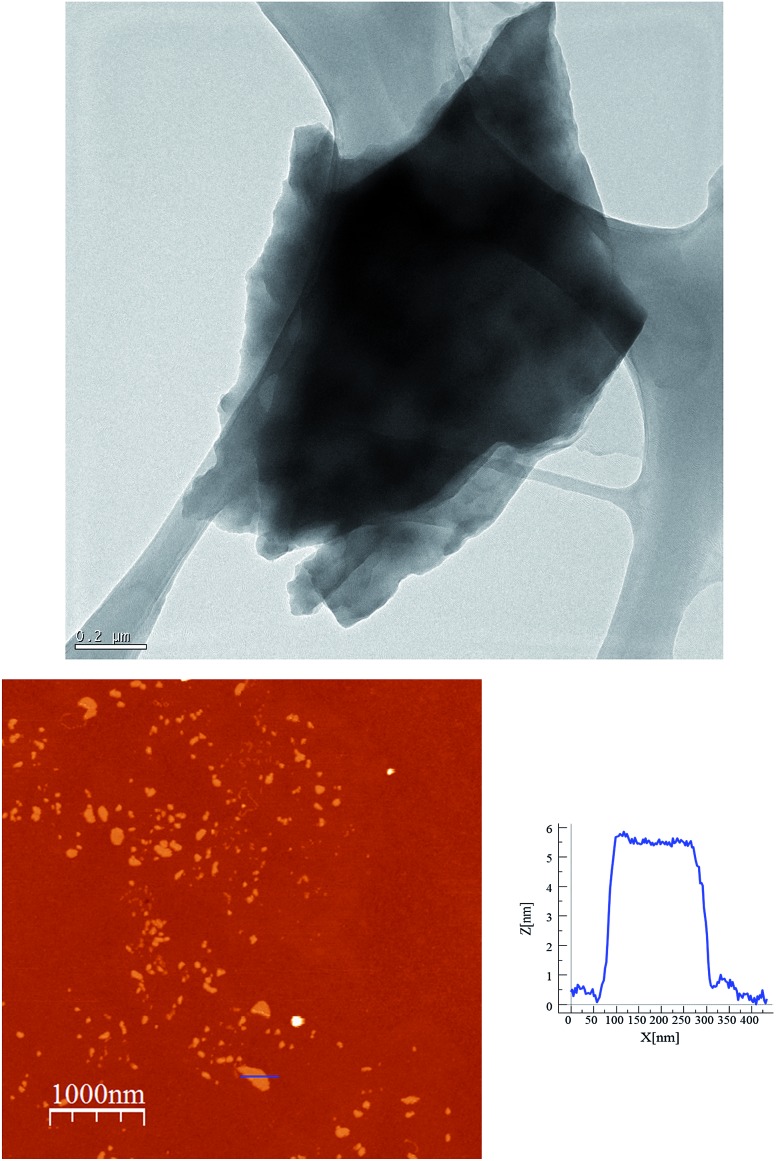
Exfoliation of **2** in ethanol. HR-TEM image of a cluster of nanosheest (top). AFM image and height profile of a nanosheet deposited on a Si substrate (bottom).

In order to check if crystals of **2** and **4** could be exfoliated by solution methods, they were immersed in acetone, ethanol or acetonitrile (1.0 mg in 1 mL) overnight and then ultrasonicated for 1 minute. In dimethylformamide, the compounds dissolved. The efficiency of the liquid exfoliation was confirmed by the observation of Tyndall light scattering of the colloidal suspensions in the two compounds (Fig. 9 for **2**) and dynamic light scattering (DLS) measurements (Fig. S11 in the ESI† and associated text). This effect was still observed in these suspensions after at least one week. For both compounds, the best results were obtained in ethanol. One drop of the ethanol suspensions was deposited on a grid for High-Resolution Transmission Electron Microscopy (HR-TEM). HR-TEM images confirm the presence of clusters of nanosheets with maximum lateral dimensions of 2 μm (Fig. 10 and S12 in the ESI†). The chemical composition is confirmed by EDS analysis with a Fe/Mn/Cr/X (X = Br for **2** and Cl for **4**) ratio of 1/1/1/6 (Fig. S12 in the ESI†). As reported for other coordination polymers, it has not been possible to observe the lattice fringes of the (*hk*0) planes by HR-TEM. That could be due to degradation by electron beam irradiation.^8^ The contrast of the TEM images indicates that the layers are very thin. AFM images of nanosheets deposited on silicon substrates are shown in Fig. 10, S13 and S14 in the ESI.† They show flakes with less well-defined borders than those obtained by mechanical exfoliation and smaller lateral dimensions (typically a few hundred nm).

These results indicate that it is possible to exfoliate hybrid coordination polymers formed by a 2D anionic network and cations inserted within or between the layers. The thicknesses of the flakes obtained by solution methods (∼5 nm) are clearly higher than those obtained by micromechanical methods, while the lateral size is significantly smaller (of the order of hundreds of nm). On the other hand, if we compare these results with those obtained in the neutral 2D coordination polymers, one observes that in the present case the solution-based exfoliation procedure is less effective than that reported in the neutral coordination polymers in which a complete delamination can be reached (with a thickness ∼1–1.5 nm).^5b,e,6,7a,8,9,10a^ This lower degree of exfoliation could be a consequence of the stronger interlayer interactions in these hybrid compounds compared with those present in neutral 2D coordination polymers. On the contrary, the micromechanical exfoliation method leads to larger and thinner flakes (∼1.5–2 nm of height) with dimensions comparable to or even larger than those obtained by solution-based methods in neutral 2D coordination polymers. Therefore, it seems that the micromechanical method is more suitable for the exfoliation of this type of hybrid coordination polymers. To our knowledge this is the first time that the Scotch tape method is used to exfoliate coordination polymers. Even more, our results indicate that this method is able to exfoliate the cationic and anionic layers together, even in salts formed by anionic layers alternating with cationic complexes (compound **4**). Hence, the presence of the cationic complexes inside the anionic anilate-based layers is not a necessary condition to achieve exfoliation. This observation suggests that this procedure is general and should be successfully used to exfoliate many other layered materials based on charged coordination polymers. In this vein, preliminary results have shown that this method can be extended to exfoliate bimetallic anionic oxalate-based networks with inserted [Fe^III^(sal_2_-trien)]^+^ complexes.

## Conclusions

Three novel magnetic compounds formed by [Fe^III^(acac_2_-trien)]^+^ or [Ga^III^(acac_2_-trien)]^+^ and anionic bimetallic coordination polymers based on the anilate ligand have been prepared and characterized. 2D anilate-based networks with a honeycomb structure have been obtained. The reduction in the size of the [Fe^III^(acac_2_-trien)]^+^ or [Ga^III^(acac_2_-trien)]^+^ complex with respect to the templating cations used in previous compounds of this type, has afforded a novel type of structure in which the cations are placed into the hexagonal channels of the 2D network instead of being placed in between the anionic layers. This has never been observed previously in oxalate or anilate-based 2D networks. This type of structure opens the way to the synthesis of a new type of multifunctional materials in which small templating cations are confined into the 1D channels defined by 2D anilate-based networks. Indeed, preliminary results with other small templating cations such as NEt_3_H^+^ show the formation of a similar structure.^26^ This close contact of the cation with the magnetic network results in an increase of the *T*_c_ (*ca.* 11 K) with respect to that of previous anilate-based compounds (*ca.* 10 K). Still, this confinement does not favour the spin-crossover of the inserted [Fe^III^(acac_2_-trien)]^+^ complexes, which remain in the high-spin state. A possible strategy to favour the spin crossover is the use of other derivatives of [Fe^III^(acac_2_-trien)]^+^ with withdrawing substituents such as Cl which favour low-spin population.^23*a*^ On the other hand, the confinement of small functional cations inside the hexagonal cavities of the honeycomb network provided by this type of structure could be a useful strategy for the introduction of other properties such as electric or proton conductivity in addition to the magnetic ordering of the anilate-based network.

Another advantage of this new magnetic network is that it can be easily exfoliated by using a micromechanical method. Thus, magnetic layers of micrometric lateral sizes and 2 nm thickness, can be isolated. In addition, we have shown that this procedure can also be used to exfoliate single layers of hybrid nature formed by an anionic magnetic layer (a bimetallic anilate network) alternating with a cationic spin-crossover layer ([Fe^III^(sal_2_-trien)]^+^). The fact that in both cases a micromechanical method can be used to exfoliate the magnetic layers represents a novelty in three aspects; (i) in contrast to the coordination polymers so far exfoliated, our compounds are not neutral 2D layered compounds (ii) they present more complex structures with two different functional sub-networks (ferrimagnetic anilate-based network and spin-crossover cation) and, (iii) a mechanical exfoliation procedure has been used for the first time to exfoliate a coordination polymer.

Hence, the exfoliation of these 2D coordination polymers provides an efficient procedure to isolate and study magnetic layers in the two dimensional limit by using local probe microscopies (such as low-temperature MFM or spin-polarized STM) and micro-SQUID measurements. In addition, the hybrid nature of the layers provides the unique opportunity to generate smart layers in which the cooperative magnetism can be tuned by the switching properties of the molecular species.
