# Supplementary material for: Graphene related magnetic materials: micromechanical exfoliation of 2D layered magnets based on bimetallic anilate complexes with inserted [FeIII(acac2-trien)]+ and [FeIII(sal2-trien)]+ molecules
Source: Chem Sci. 2015 May 26;6(8):4665–73. doi: 10.1039/c5sc00957j (PMC5500854; doi:10.1039/c5sc00957j)
Supplement: Supplementary file 1 [file SC-006-C5SC00957J-s001.pdf]

Electronic Supplementary Material (ESI) for Chemical Science.
This journal is © The Royal Society of Chemistry 2015

## Experimental Section

**General Remarks:** $[Fe^{III}(acac_2\text{-trien})]PF_6$ and $[Ga^{III}(acac_2\text{-trien})]PF_6$ were prepared according to literature methods.[1] $[(n\text{-Bu})_4N]_3[Cr^{III}(X_2An)_3]$ (X=Cl, Br) were prepared as described in the literature.[2] All other materials and solvents were commercially available and used without further purification.

**Syntheses:** $[Fe^{III}(acac_2\text{-trien})][Mn^{II}Cr^{III}(Cl_2An)_3]\cdot(CH_3CN)_2$ (**1**), $[Fe^{III}(acac_2\text{-trien})][Mn^{II}Cr^{III}(Br_2An)_3]\cdot(CH_3CN)_2$ (**2**) and $[Ga^{III}(acac_2\text{-trien})][Mn^{II}Cr^{III}(Br_2An)_3]\cdot(CH_3CN)_2$ (**3**) were obtained by slow diffusion of two solutions. The first solution was prepared by dissolving $MnCl_2\cdot 4H_2O$ (12 mg, 0.06 mmol) and $[Fe^{III}(acac_2\text{-trien})](PF_6)$ or $[Ga^{III}(acac_2\text{-trien})](PF_6)$ (0.06 mmol) in 6 mL of a 9:1 chloroform/methanol mixture (**1**) or 9:1 dichloromethane/methanol mixture (**2** and **3**). The second solution was obtained by dissolving $[NBu_4]_3[Cr^{III}(Cl_2An)_3]$ (84 mg, 0.06 mmol) (for **1**) or $[NBu_4]_3[Cr^{III}(Br_2An)_3]$ (100 mg, 0.06 mmol) (for **2** and **3**) in acetonitrile (6 mL). After 4 weeks, black prismatic single crystals suitable for X-ray crystal analysis were obtained. Yields: 10 % (**1**), 10 % (**2**) and 8 % (**3**). These yields could be improved by diffusing the two solutions for longer times. Anal. Calc. for $C_{38}H_{34}Cl_6CrFeMnN_6O_{14}$ (**1**): C, 38.9; H, 2.9; N, 7.2 %. Found: C, 35.3; H, 2.6; N, 4.6 %. Calc. for $C_{38}H_{34}Br_6CrFeMnN_6O_{14}$ (**2**): C, 31.7; H, 2.4; N, 5.8 %. Found: C, 31.6; H, 1.7; N, 5.4 %. Calc. for $C_{38}H_{34}Br_6CrGaMnN_6O_{14}$ (**3**): C, 31.4; H, 2.4; N, 5.8 %. Found: C, 28.9; H, 2.1; N, 4.2 %.

**Structural Characterization:** Single crystals of all compounds were mounted on glass fibres using a viscous hydrocarbon oil to coat the crystal and then transferred directly to the cold nitrogen stream for data collection. All reflection data were collected at 120 K on a Supernova diffractometer equipped with a graphite-monochromated Enhance (Mo) X-ray Source ($\lambda$ = 0.71073 Å). The CrysAlisPro program, Oxford Diffraction Ltd., was used for unit cell determinations and data reduction. Empirical absorption correction was performed using spherical harmonics, implemented in the SCALE3 ABSPACK scaling algorithm. Crystal structures were solved by direct methods with the SIR97 program,[3] and refined against all $F^2$ values with the SHELXL-2013 program,[4] using the WinGX graphical user interface.[5] All non-hydrogen atoms were refined anisotropically, and hydrogen atoms were placed in calculated positions and refined isotropically with a riding model. The details of data collection and structure refinements are provided in Table 1. 0.5 mm glass capillaries were filled with polycrystalline samples of **2** and **3** and mounted and aligned on a Empyrean PANalytical powder diffractometer, using Cu$K\alpha$ radiation ($\lambda$ = 1.54177 Å). A total of 3 or 5 scans were collected at room temperature in the $2\theta$ range 5-40°.

**Physical Measurements:** Magnetic measurements were performed with a Quantum Design MPMS-XL-5 SQUID magnetometer in the 2 to 300 K temperature range with an applied magnetic field of 0.1

T on polycrystalline samples of **1** (12 mg), **2** (4.9 mg) and **3** (7.2 mg). The AC measurements were performed in the temperature range 2-15 K at different frequencies with an oscillating magnetic field of 0.395 mT. The magnetization and hysteresis studies were performed between 5 and -5 K T with a scan speed of 0.22 T/min, cooling the samples at zero field. C, H and N elemental analyses were measured on a CE Instruments EA 1110 CHNS Elemental analyzer. The Fe/Ga/Mn/Cr/Cl/Br ratios and scanning electron microscopy (SEM) images were obtained with a Philips ESEM X230 scanning electron microscope equipped with an EDAX DX-4 microsonde.

**Characterization of the nanosheets:** High-resolution transmission electronic microscopy (TEM) measurements were performed on a TECNAI-F20 microscope operating at 200 kV. High-resolution SEM images were measured on a Hitachi S-4800 field emission scanning electron microscope. Comercial atomic force microscopes (Multimode SPM by Veeco and Nanotec Cervantes Full Mode AFM by Nanotec Electrónica S.L.) were employed for surface sample characterization. AFM was operated in tapping mode. Sharp silicon probes were purchased from Nanosensors (PPP-NCH-W; force constant: 10-130 N/m; resonance frequency: 204-497 kHz). Scan rate was adjusted during the scanning of each image (usually below 1 Hz, 512 samples/line). AFM images were processed by using WSxM.[6] DLS measurements were performed in a Malvern ZetaSizer ZS.

| | Mn-O distances | Cr-O distances |
|---|---|---|
| **1** | 2.172(2)-2.186(2) Å | 1.970(2)-1.974(2) Å |
| **2** | 2.167(5)-2.177(5) Å | 1.973(5)-1.985(5) Å |
| **3** | 2.175(4)-2.183(4) Å | 1.972(4)-1.983(4) Å |

As usual for this type of complexes, the Fe/Ga–O distances are the shortest ones (1.939(3) Å for **1**, 1.949(5) Å for **2** and 1.949(5) for **3**), the Fe–N(amine) distances are the longest ones (2.172(3) Å for **1**, 2.186(6) Å for **2** and 2.140(5) Å for **3**), whereas the Fe–N(imine) bonds are intermediate (2.110(3) Å for **1**, 2.113(6) Å for **2** and 2.041(6) for **3**).

**Table 1.** Crystallographic data for compounds **1, 2** and **3**.

| Compound | **1** | **2** | **3** |
|---|---|---|---|
| Empirical formula | $C_{38}H_{34}Cl_6CrFeMnN_6O_{14}$ | $C_{38}H_{34}Br_6CrFeMnN_6O_{14}$ | $C_{38}H_{34}Br_6CrGaMnN_6O_{14}$ |
| Formula weight | 1174.20 | 1440.96 | 1454.79 |
| Crystal colour | Black | Black | Purple |
| Crystal size | 0.06×0.06×0.05 | 0.06×0.05×0.04 | 0.06×0.04×0.03 |
| Temperature (K) | 120(2) | 120(2) | 120(2) |
| Wavelength (Å) | 0.71073 | 0.71073 | 0.71073 |
| Crystal system, *Z* | Monoclinic, 4 | Monoclinic, 4 | Monoclinic, 4 |
| Space group | *C* 2/*c* | *C* 2/*c* | *C* 2/*c* |
| *a* (Å) | 14.2177(3) | 14.1914(5) | 14.1613(4) |
| *b* (Å) | 22.9278(5) | 23.0061(7) | 23.0839(6) |
| *c* (Å) | 14.5428(4) | 14.7880(5) | 14.8676(5) |
| α (°) | 90 | 90 | 90 |
| ß (°) | 105.118(2) | 104.703(3) | 104.296(3) |
| γ (°) | 90 | 90 | 90 |
| $V$ (Å$^3$) | 4576.60(19) | 4670.0(3) | 4709.7(2) |
| $\rho_{calc}$ (Mg/m$^3$) | 1.704 | 2.049 | 2.052 |
| μ(Mo$_{K\alpha}$) (mm$^{-1}$) | 1.240 | 6.006 | 6.220 |
| $\theta$ range (°) | 3.028-26.394 | 2.968-26.405 | 2.969-26.380 |
| Reflns collected | 33850 | 35510 | 59295 |
| Independent reflns ($R_{int}$) | 4679(0.0742) | 4797(0.0779) | 4825(0.1654) |
| L. S. parameters, *p*/ restraints, *r* | 305 / 0 | 293 / 0 | 305 / 0 |
| $R1(F)$,[a] $I > 2\sigma(I)$ | 0.0441 | 0.0616 | 0.0543 |
| $wR2(F^2)$,[b] all data | 0.1197 | 0.1765 | 0.1394 |
| $S(F^2)$,[c] all data | 1.062 | 1.082 | 1.003 |

[a] $R1(F) = \Sigma||F_O|-|F_C||/\Sigma|F_O|$; [b] $wR2(F^2) = [\Sigma w(F_O{}^2-F_C{}^2)^2/\Sigma wF_O{}^4]^{½}$; [c] $S(F^2) = [\Sigma w(F_O{}^2-F_C{}^2)^2/(n + r - p)]^{½}$

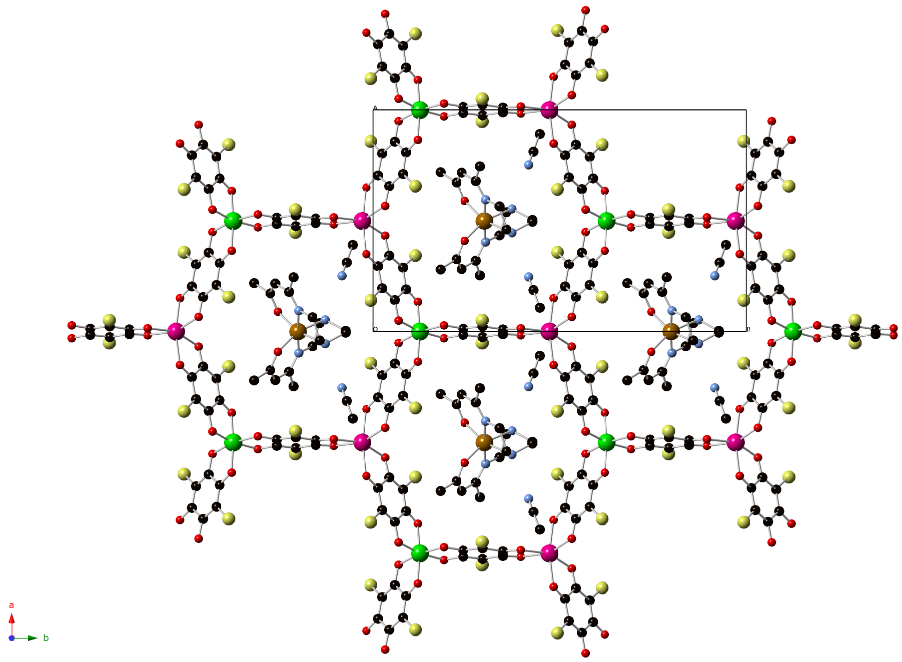

(a)

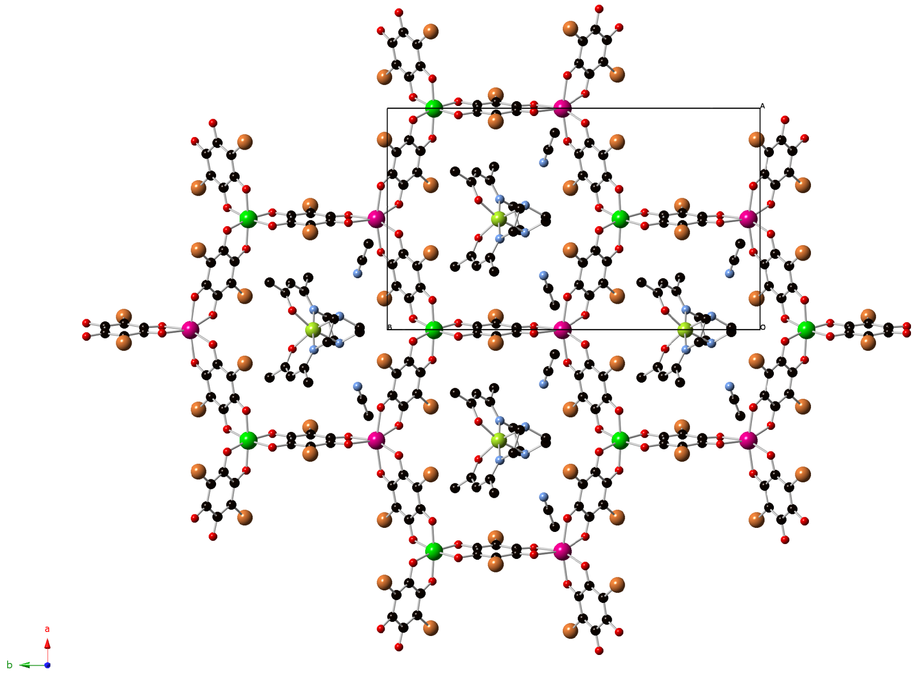

(b)

**Figure S1.** Projection of **1** (a) and **3** (c) in the *ab* plane (Fe (brown), Ga (olive green), Cr (green), Mn (pink) C (black), N (blue), O (red), Cl (yellow), Br (orange)). Hydrogen atoms have been omitted for clarity.

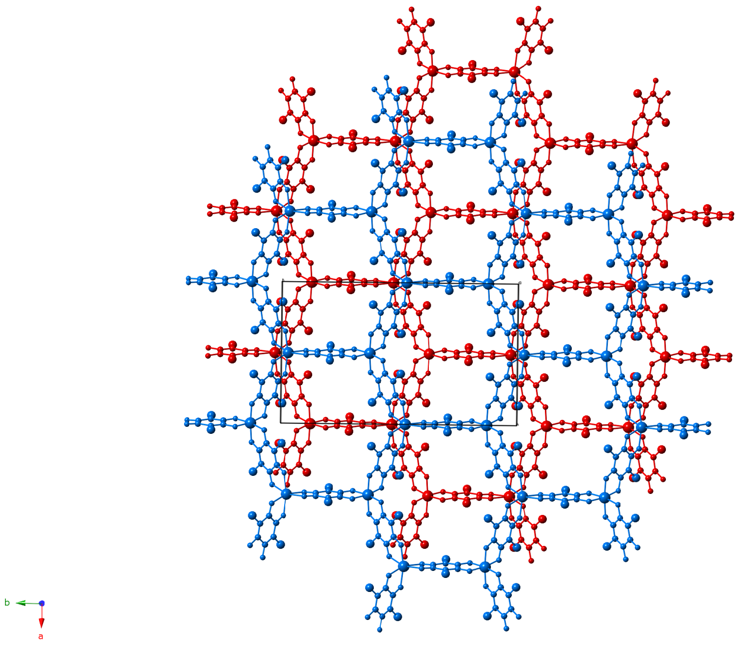


(a)

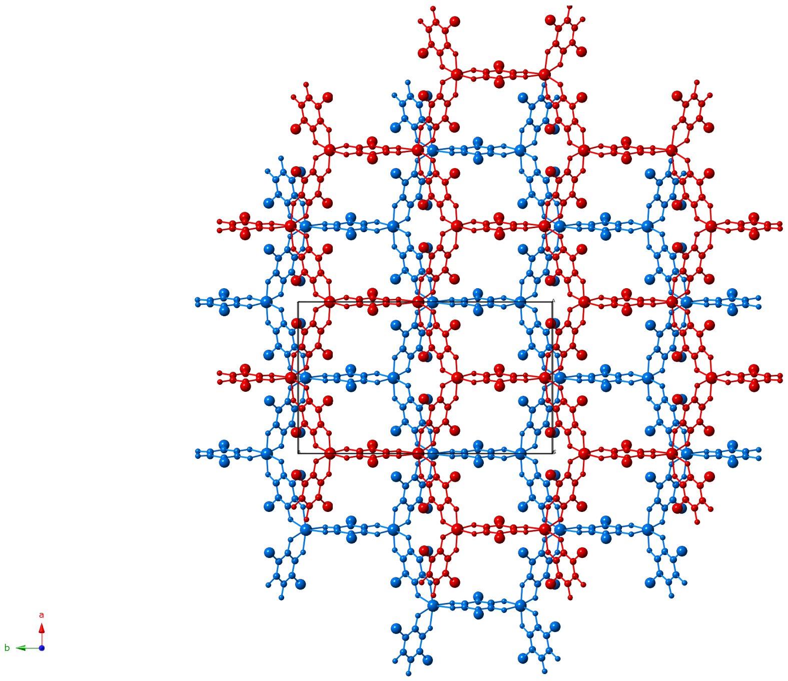


(b)

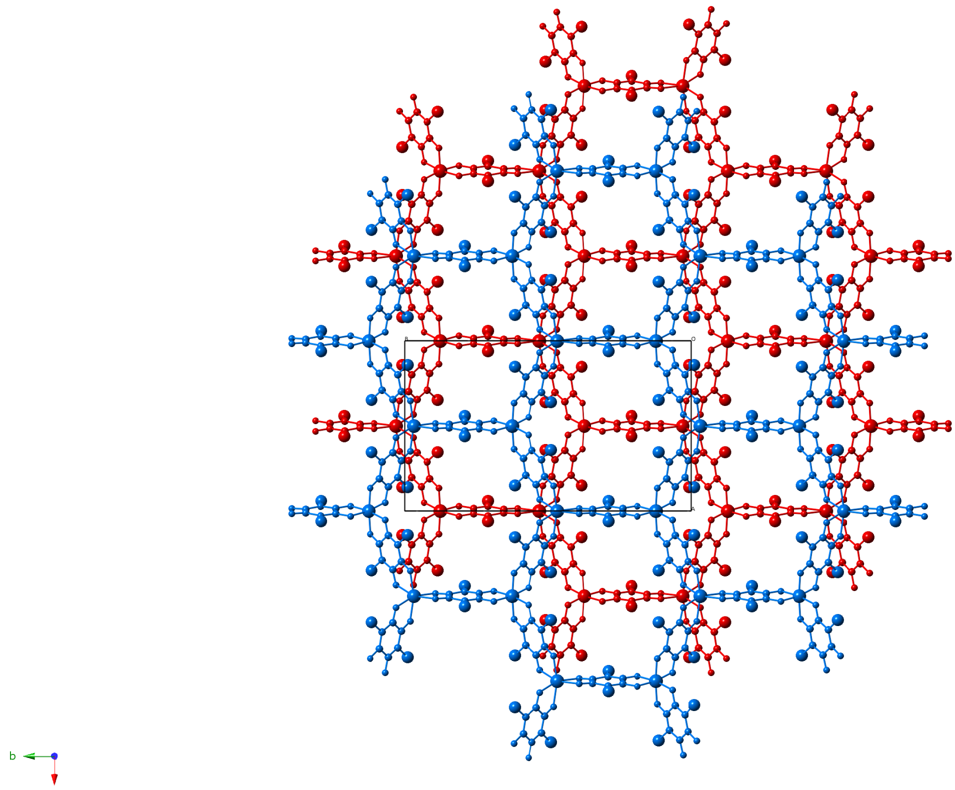


(c)

**Figure S2.** Projection, perpendicular to the layers, of two consecutive anionic layers of **1** (a), **2** (b) and **3** (c) showing their alternate packing.

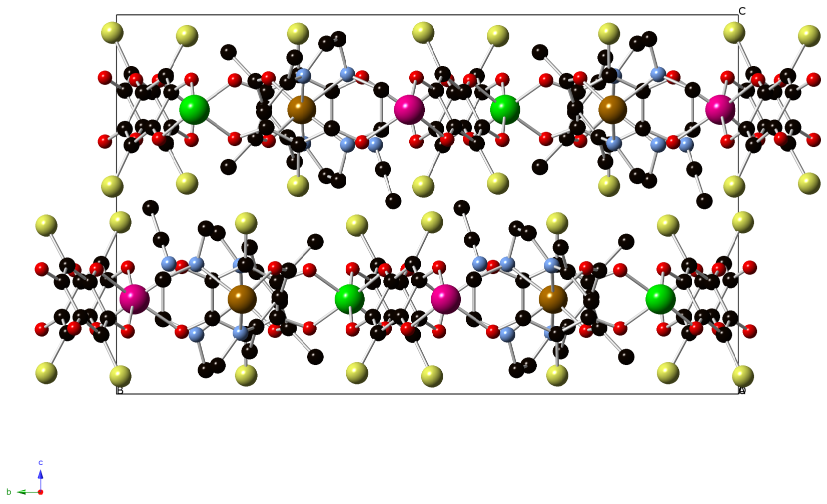

(a)

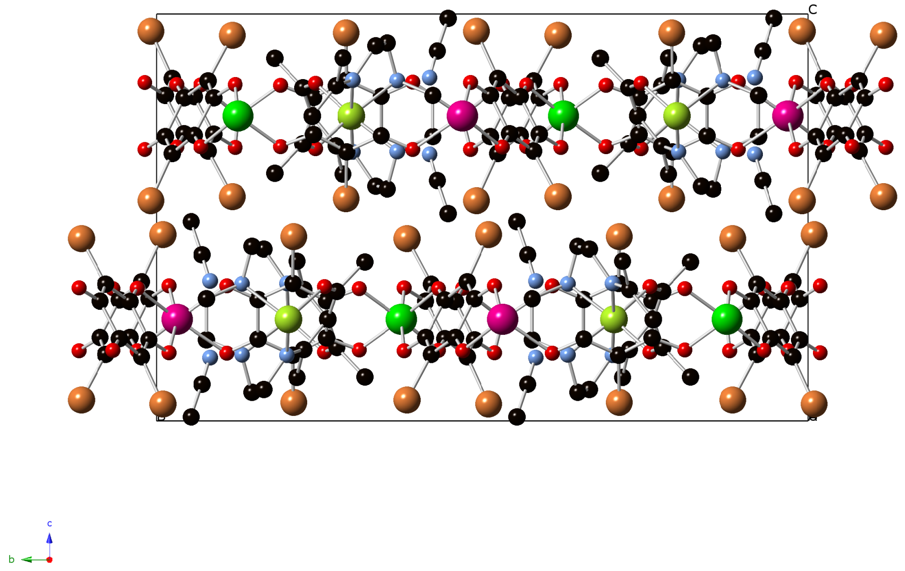

(b)

**Figure S3.** Projection of **1** (a) and **3** (b) in the *bc* plane (Fe (brown), Ga (olive green), Cr (green), Mn (pink) C (black), N (blue), O (red), Cl (yellow), Br (orange)). Hydrogen atoms have been omitted for clarity.

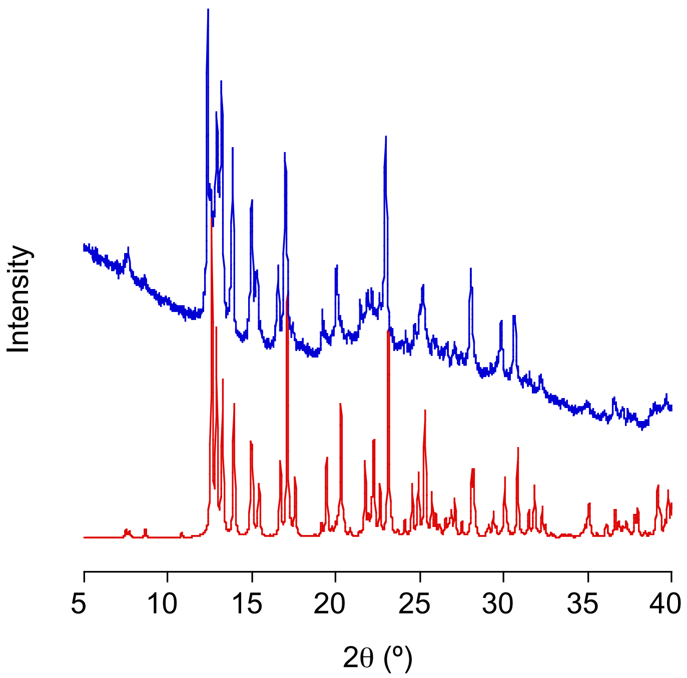
Intensity
5
10
15
20
25
30
35
40
2θ (º)

(a)

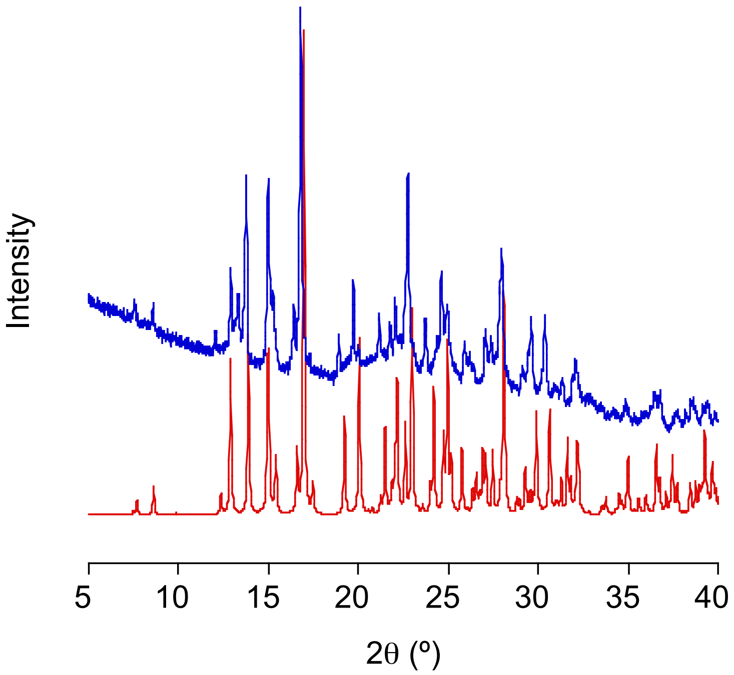
Intensity
5
10
15
20
25
30
35
40
2θ (º)

(b)

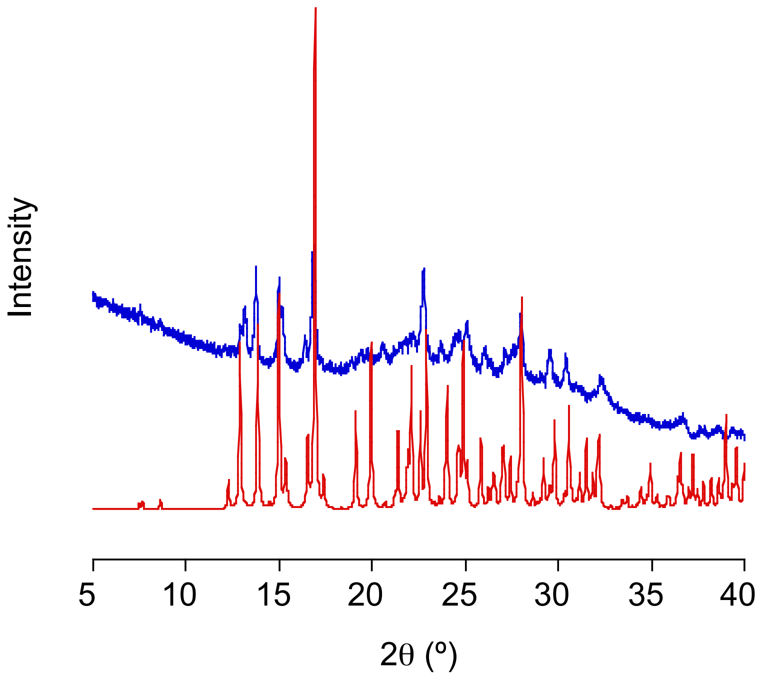


(c)

**Figure S4.** Powder X-ray diffraction pattern (top) and simulated pattern (bottom) of **1** (a), **2** (b) and **3** (c).

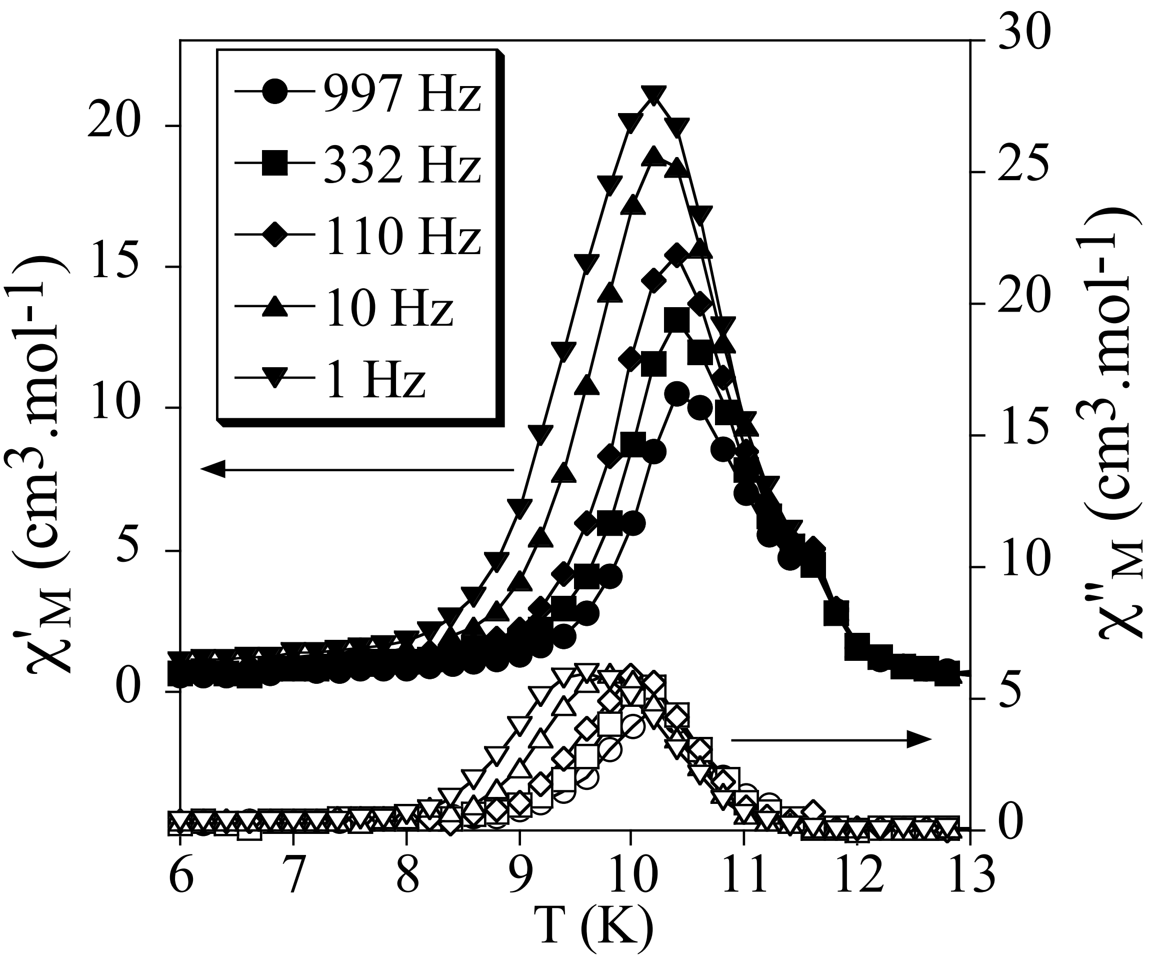


**Figure S5.** Temperature dependence of the in-phase AC susceptibility (χ') (filled symbols) and the out-of-phase AC susceptibility (χ") of **3**.

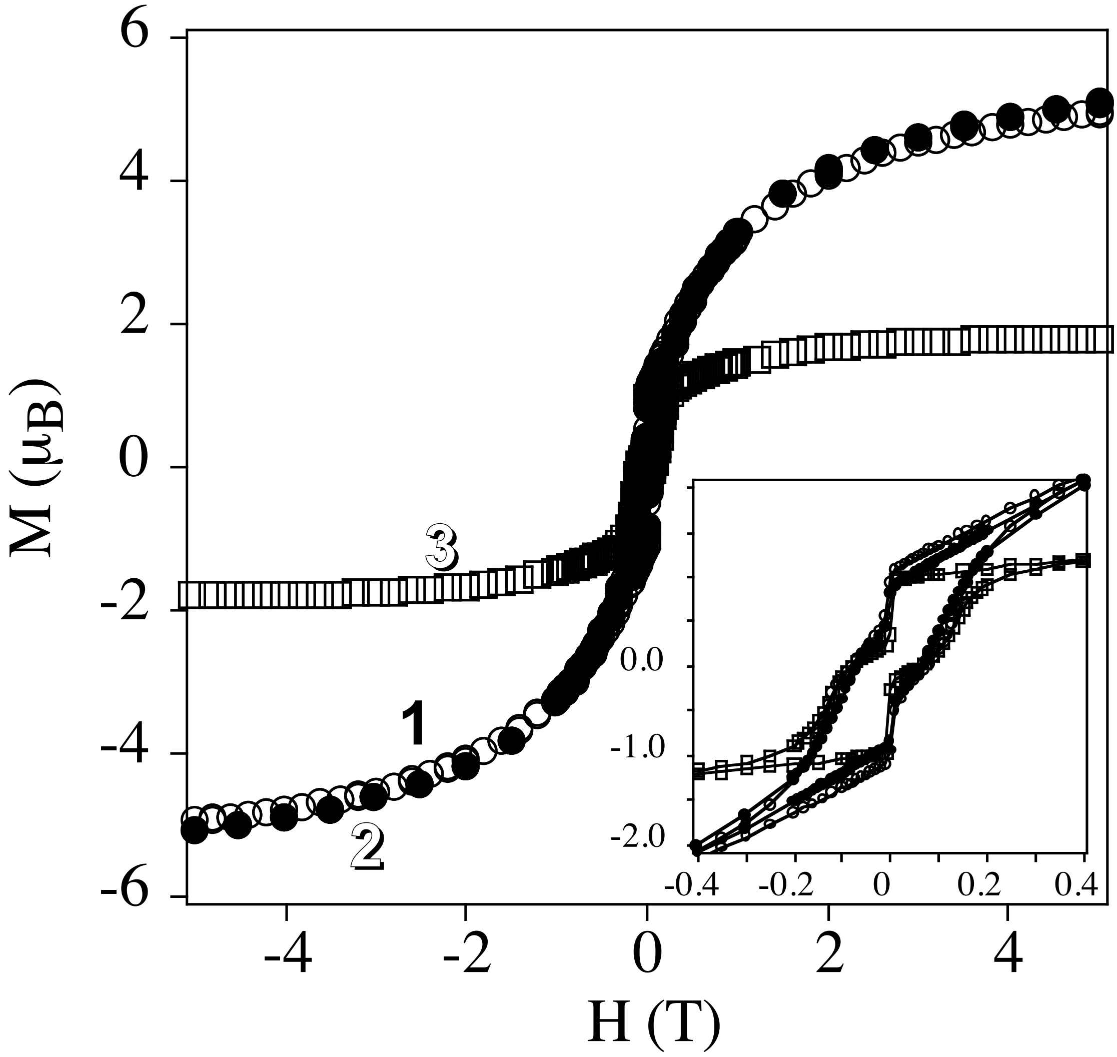


**Figure S6.** Isothermal magnetization at 2 K of **1** (full circles), **2** (empty circles) and **3** (empty squares).

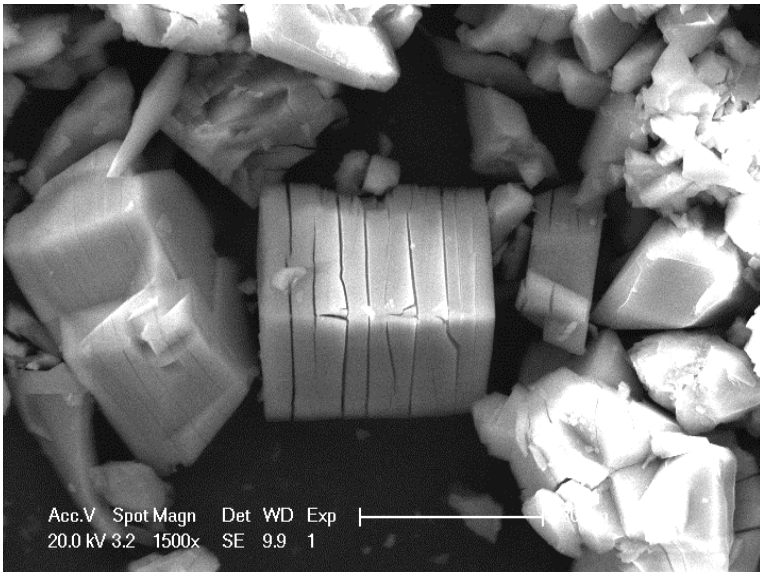


**Figure S7.** SEM image of bulk crystals of **2**.

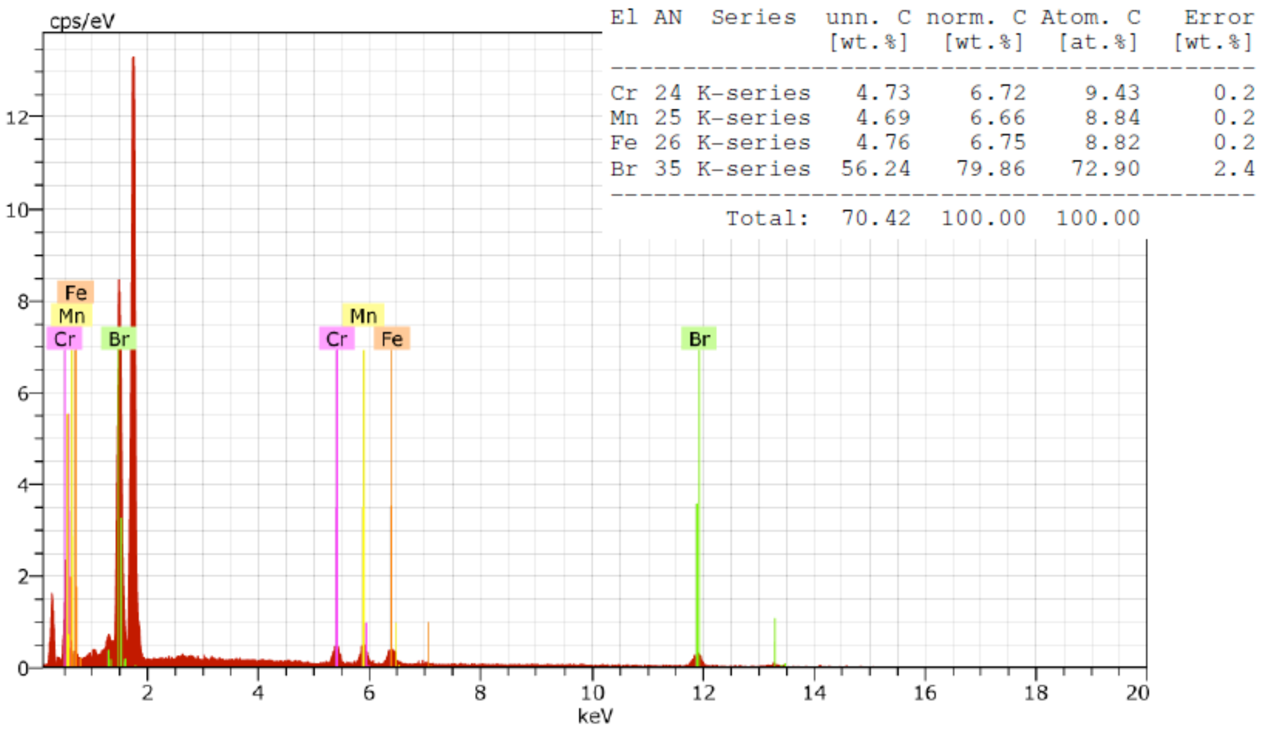


| El | AN | Series | unn. C [wt.%] | norm. C [wt.%] | Atom. C [at.%] | Error [wt.%] |
|---|---|---|---|---|---|---|
| Cr | 24 | K-series | 4.73 | 6.72 | 9.43 | 0.2 |
| Mn | 25 | K-series | 4.69 | 6.66 | 8.84 | 0.2 |
| Fe | 26 | K-series | 4.76 | 6.75 | 8.82 | 0.2 |
| Br | 35 | K-series | 56.24 | 79.86 | 72.90 | 2.4 |
| | | Total: | 70.42 | 100.00 | 100.00 | |

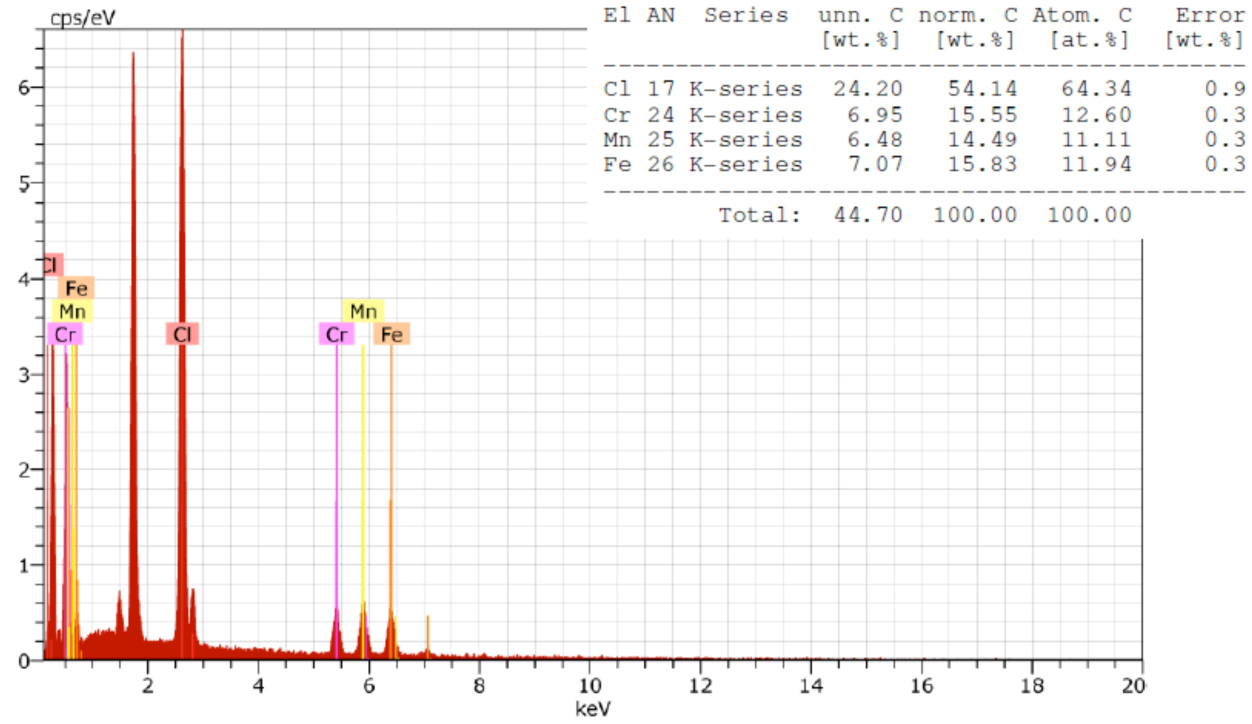


| El | AN | Series | unn. C [wt.%] | norm. C [wt.%] | Atom. C [at.%] | Error [wt.%] |
|---|---|---|---|---|---|---|
| Cl | 17 | K-series | 24.20 | 54.14 | 64.34 | 0.9 |
| Cr | 24 | K-series | 6.95 | 15.55 | 12.60 | 0.3 |
| Mn | 25 | K-series | 6.48 | 14.49 | 11.11 | 0.3 |
| Fe | 26 | K-series | 7.07 | 15.83 | 11.94 | 0.3 |
| | | Total: | 44.70 | 100.00 | 100.00 | |

**Figure S8.** Microanalysis recorded during the SEM characterization of flakes of **2** (top) and **4** (bottom) obtained by mechanical exfoliation on a 285 nm $SiO_2$/Si substrate.

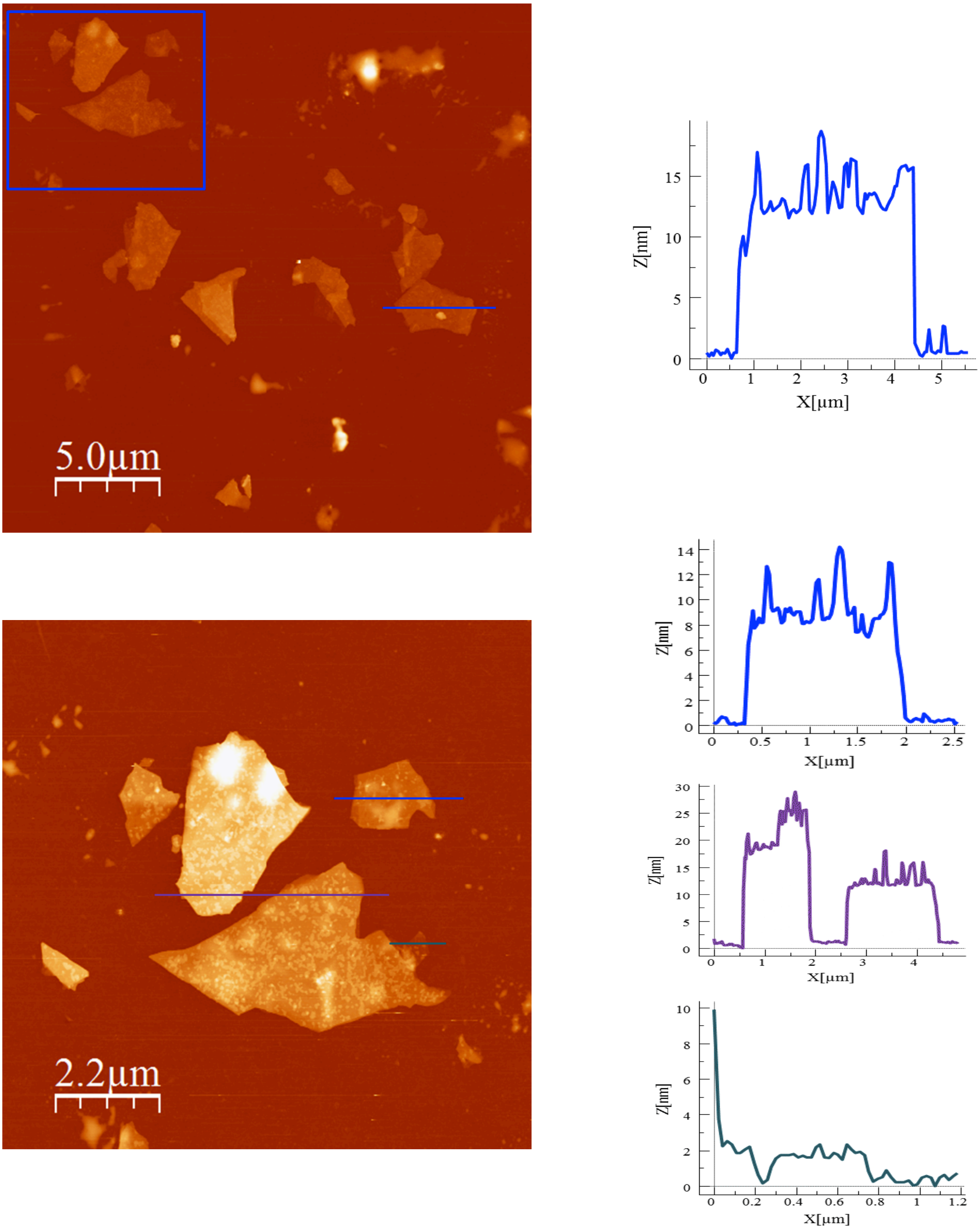


**Figure S9.** AFM images and height profiles of flakes of **2** obtained by mechanical exfoliation on a 285 nm $SiO_2/Si$ substrate.

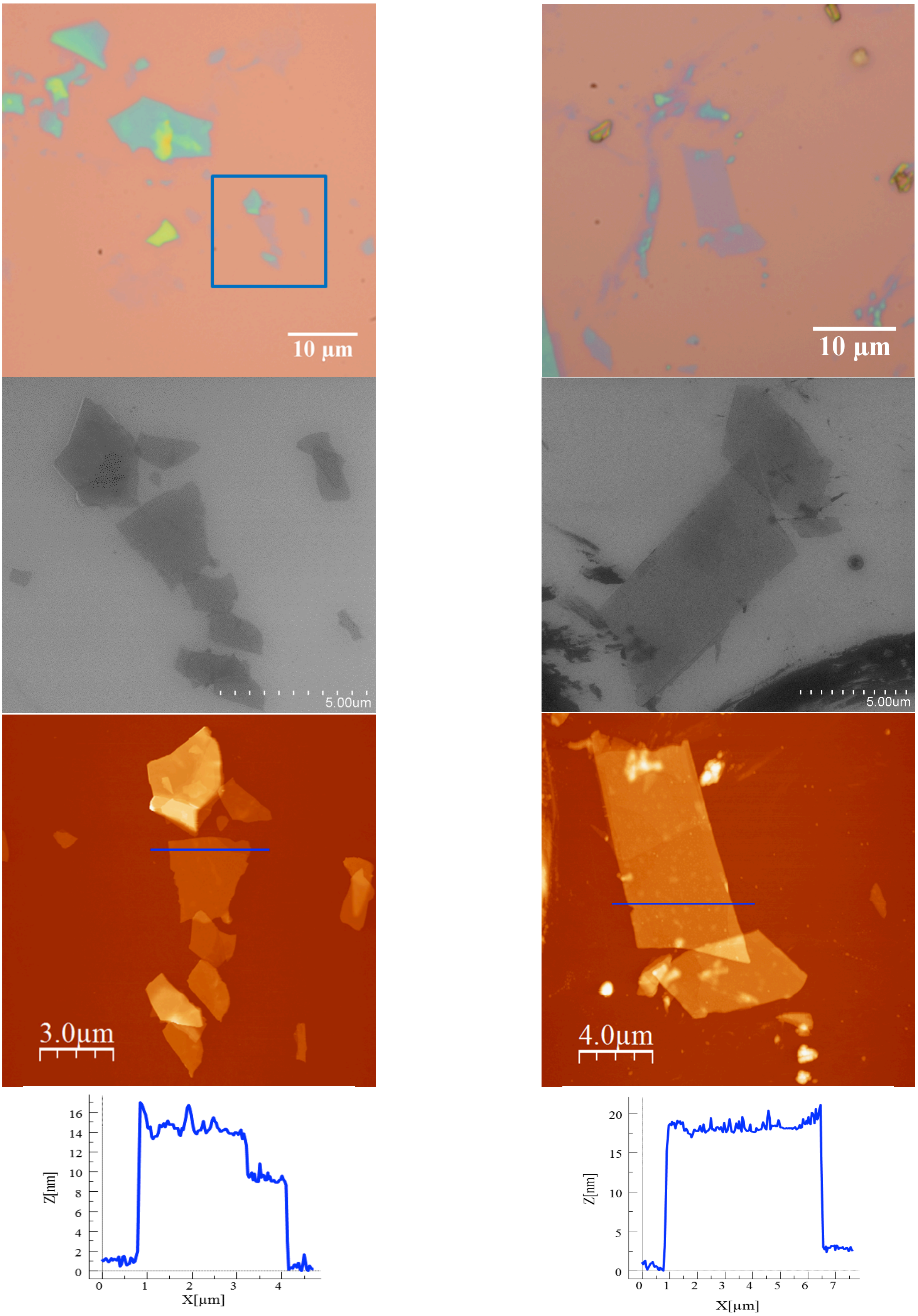


**Figure S10.** Optical microscopy images (top), SEM images (middle) and AFM images with height profiles (bottom) of flakes of **4** obtained by mechanical exfoliation on a 285 nm $SiO_2$/Si substrate taken in the same zone.

Acetone

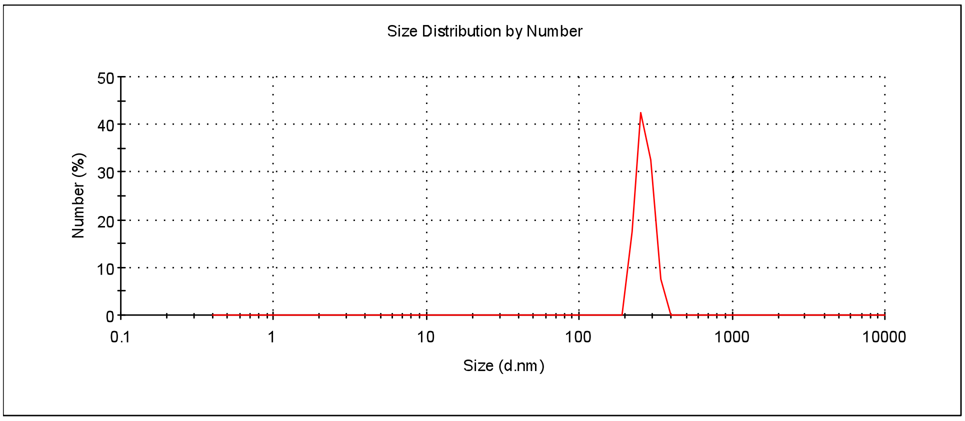


Ethanol

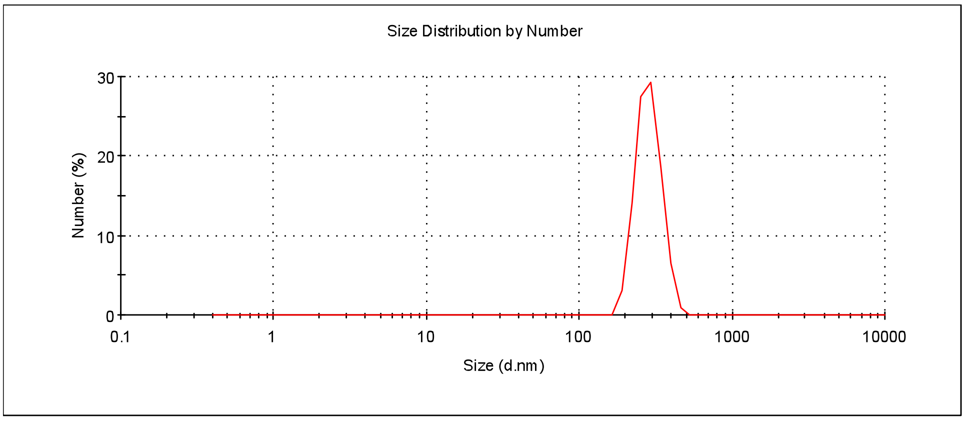


Acetonitrile

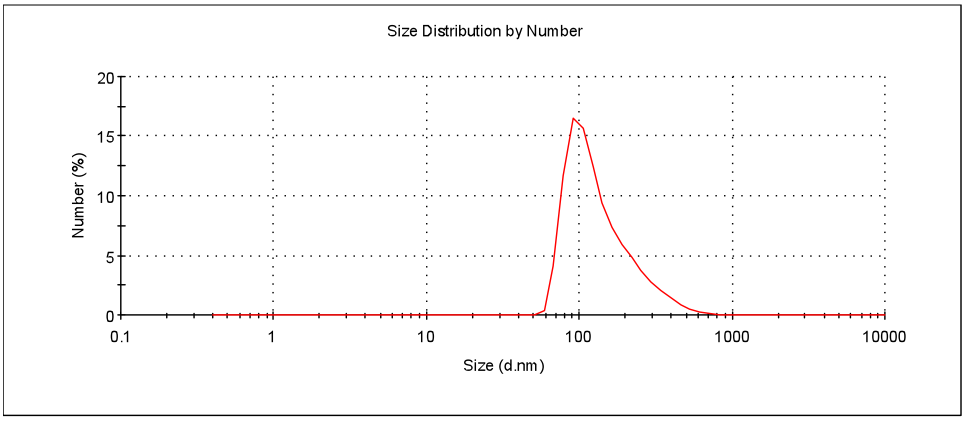


The size of the exfoliated layers in the three suspensions of **2**, estimated from dynamic light scattering (DLS), shows a distribution centred around 270 nm in acetone, 290 nm in ethanol and 145 nm in acetonitrile.

**Figure S11.** DLS measurements of suspensions of crystals of **2** (1.0 mg in 1 mL) after immersing in acetone (top), ethanol (middle) or acetonitrile (bottom) overnight and then ultrasonicating for 1 minute.

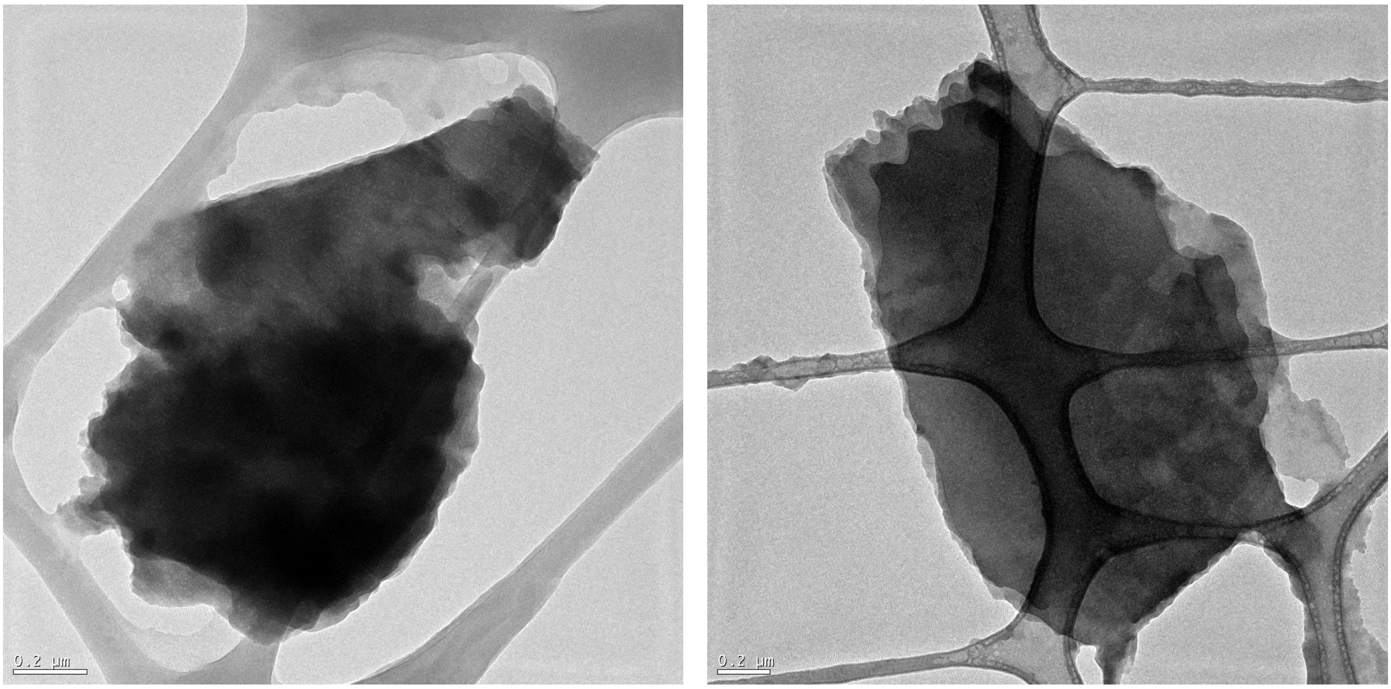


| Element | Weight % | Atomic % |
|---|---|---|
| CrK | 8.1 | 11.1 |
| MnK | 9.6 | 12.4 |
| FeK | 9.6 | 12.2 |
| BrK | 72.6 | 64.4 |
| Total | 100.0 | 100.0 |

| Element | Net Inte. | Backgrd | Inte. Error | P/B |
|---|---|---|---|---|
| CrK | 13.86 | 1.12 | 4.09 | 12.38 |
| MnK | 16.14 | 1.12 | 3.76 | 14.41 |
| FeK | 15.72 | 1.20 | 3.83 | 13.10 |
| BrK | 62.22 | 0.76 | 1.81 | 81.87 |

| Element | Weight % | Atomic % |
|---|---|---|
| ClK | 54.0 | 64.3 |
| CrK | 14.4 | 11.7 |
| MnK | 13.9 | 10.7 |
| FeK | 17.6 | 13.3 |
| Total | 100.0 | 100.0 |

| Element | Net Inte. | Backgrd | Inte. Error | P/B |
|---|---|---|---|---|
| ClK | 120.86 | 2.30 | 1.31 | 52.55 |
| CrK | 21.42 | 1.12 | 3.21 | 19.13 |
| MnK | 20.26 | 1.40 | 3.35 | 14.47 |
| FeK | 25.14 | 1.80 | 3.02 | 13.97 |

**Figure S12.** HR-TEM images and EDS analysis of flakes of **2** (left) and **4** (right) obtained by solvent-mediated exfoliation in ethanol and deposited on a TEM grid.

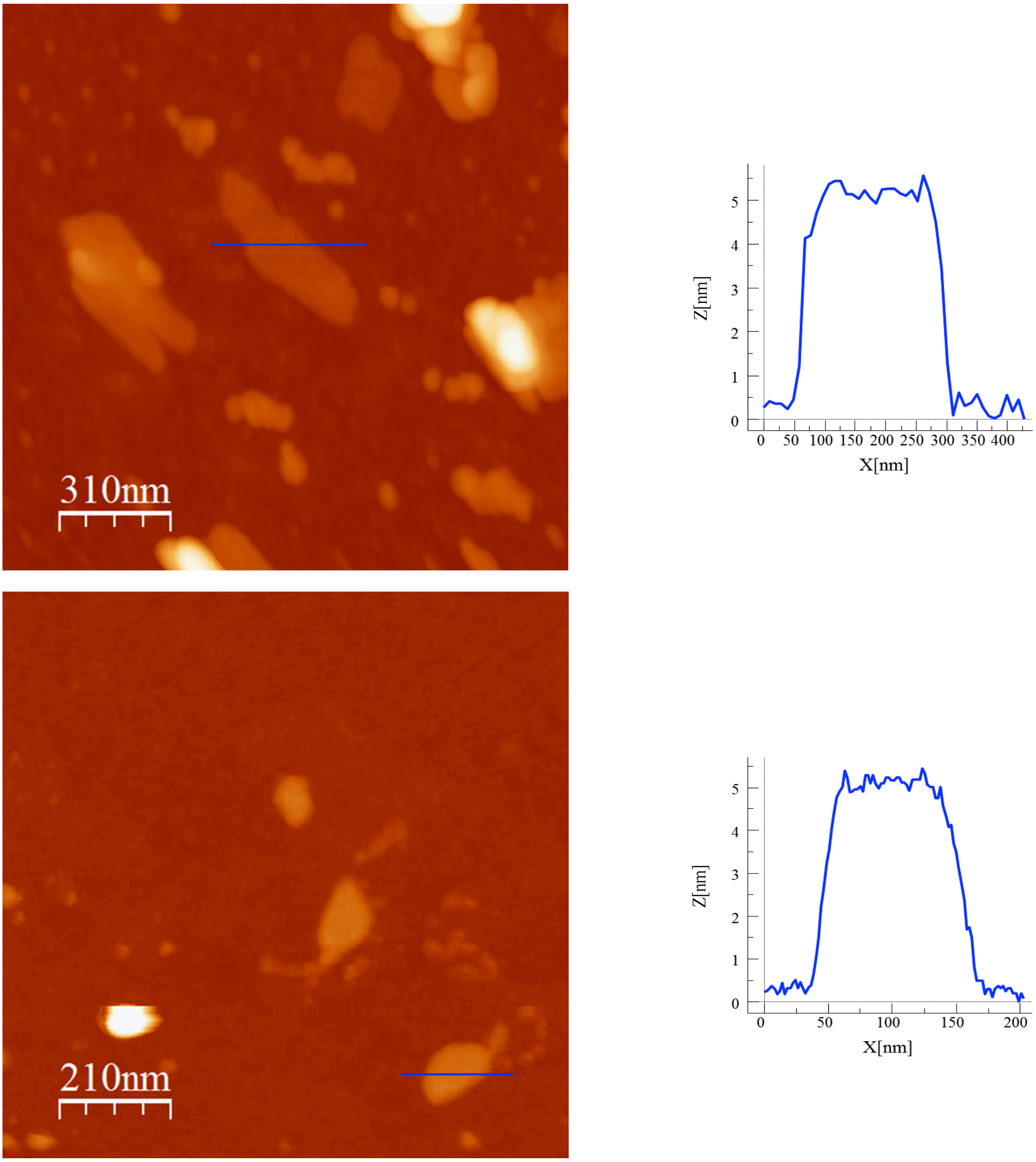


**Figure S13.** AFM images and height profiles of nanosheets of **2** exfoliated in ethanol and deposited on a Si substrate.

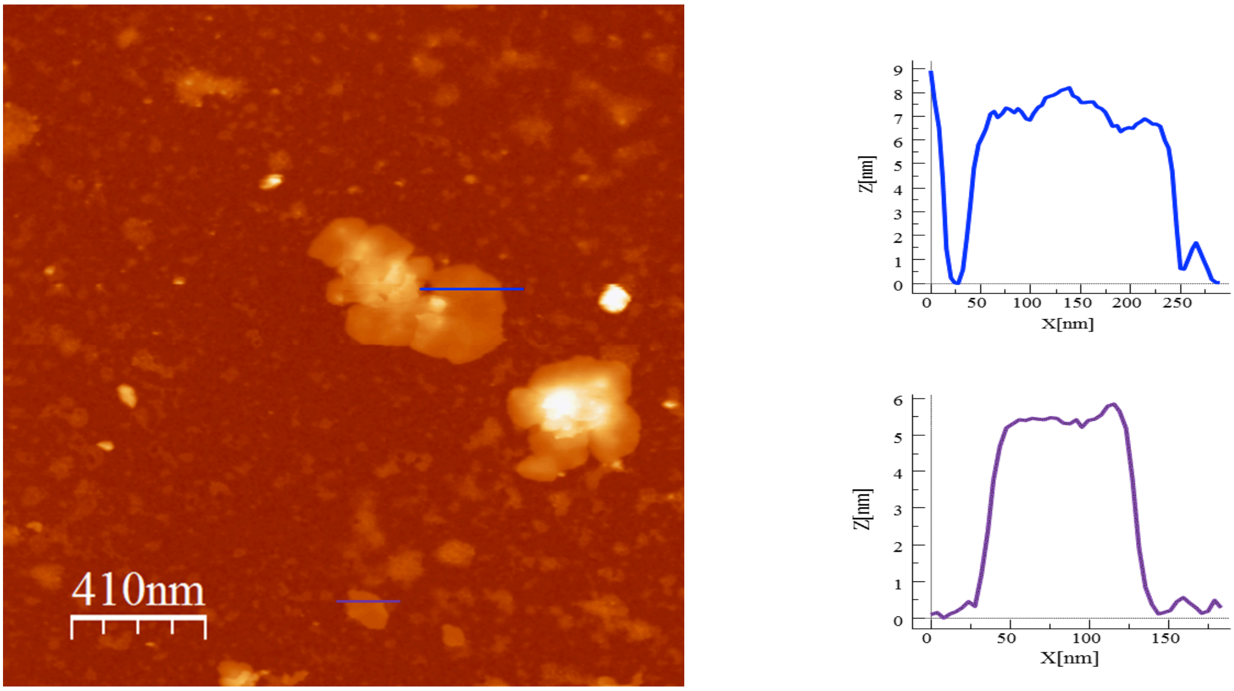


**Figure S14.** AFM images and height profiles of nanosheets of **4** exfoliated in ethanol and deposited on a Si substrate.

---

[1] E. V. Dose, K. M. M. Murphy and L. J. Wilson, *Inorg. Chem.*, 1976, **15**, 2622.

[2] A. Abhervé, M. Clemente-León, E. Coronado, C. J. Gómez-García and M. Verneret, *Inorg. Chem.*, 2014, **53**, 12014.

[3] A. Altomare, M. C. Burla, M. Camalli, G. L. Cascarano, C. Giacovazzo, A. Guagliardi, A. G. G. Moliterni, G. Polidori and R. Spagna, *J. Appl. Cryst.*, 1999, **32**, 115.

[4] G. M. Sheldrick, *Acta Cryst.*, 2008, **A64,** 112.

[5] L. J. Farrugia, *J. Appl. Cryst.*, 2012, **45**, 849.

[6] I. Horcas, R. Fernández, J. M. Gómez-Rodríguez, J. Colchero, J. Gómez-Herrero and A. M. Baro, *Rev. Sci. Instrum.*, 2007, **78**, 013705.
